# New Discorhabdin Alkaloids from the Antarctic Deep-Sea Sponge *Latrunculia biformis*

**DOI:** 10.3390/md17080439

**Published:** 2019-07-25

**Authors:** Fengjie Li, Christian Peifer, Dorte Janussen, Deniz Tasdemir

**Affiliations:** 1 GEOMAR Centre for Marine Biotechnology (GEOMAR-Biotech), Research Unit Marine Natural Products Chemistry, GEOMAR Helmholtz Centre for Ocean Research Kiel, Am Kiel-Kanal 44, 24106 Kiel, Germany; 2 Pharmaceutical Chemistry, Kiel University, Gutenbergstraße 76, 24118 Kiel, Germany; 3 Senckenberg Research Institute and Natural History Museum, Senckenberganlage 25, D-60325 Frankfurt, Germany; 4 Faculty of Mathematics and Natural Sciences, Kiel University, Christian-Albrechts-Platz 4, 24118 Kiel, Germany

**Keywords:** *Latrunculia*, Antarctica, deep-sea sponge, molecular networking, molecular docking, discorhabdin

## Abstract

The sponge genus *Latrunculia* is a prolific source of discorhabdin type pyrroloiminoquinone alkaloids. In the continuation of our research interest into this genus, we studied the Antarctic deep-sea sponge *Latrunculia biformis* that showed potent in vitro anticancer activity. A targeted isolation process guided by bioactivity and molecular networking-based metabolomics yielded three known discorhabdins, (−)-discorhabdin L (**1**), (+)-discorhabdin A (**2**), (+)-discorhabdin Q (**3**), and three new discorhabdin analogs (−)-2-bromo-discorhabdin D (**4**), (−)-1-acetyl-discorhabdin L (**5**), and (+)-1-octacosatrienoyl-discorhabdin L (**6**) from the MeOH-soluble portion of the organic extract. The chemical structures of **1**–**6** were elucidated by extensive NMR, HR-ESIMS, FT-IR, [α]_D_, and ECD (Electronic Circular Dichroism) spectroscopy analyses. Compounds **1**, **5,** and **6** showed promising anticancer activity with IC_50_ values of 0.94, 2.71, and 34.0 µM, respectively. Compounds **1**–**6** and the enantiomer of **1** ((+)-discorhabdin L, **1e**) were docked to the active sites of two anticancer targets, topoisomerase I-II and indoleamine 2,3-dioxygenase (IDO1), to reveal, for the first time, the binding potential of discorhabdins to these proteins. Compounds **5** and **6** are the first discorhabdin analogs with an ester function at C-1 and **6** is the first discorhabdin bearing a long-chain fatty acid at this position. This study confirms *Latrunculia* sponges to be excellent sources of chemically diverse discorhabdin alkaloids.

## 1. Introduction

*Latrunculia* species are cold-adapted sponges commonly found in the coastlines of the southern hemisphere [1,2,3]. The genus *Latrunculia* has proven to be a prolific source of structurally intriguing compounds from different classes, such as norsesterterpenes [4,5], callipeltins [6,7], and various types of pyrroloiminoquinone alkaloids [8,9,10]. Discorhabdins represent a large and unique subclass of pyrroloiminoquinone alkaloids that have been associated with the chemical defense and greenish to brownish coloration of the sponge [11,12]. Discorhabdins exhibit strong anticancer activity against many cancer types, such as human colon cancer, adenocarcinoma, and leukemia [13,14,15]. However, the mechanism of their anticancer action has been poorly studied. Indeed, only the farnesyltransferase enzyme [16] and hypoxia-inducible factor 1α (HIF-1α) and transcriptional coactivator p300 interaction [17] have been shown as potential targets of discorhabdins.

As part of our research interest into deep-sea *Latrunculia* sponges from Antarctica [18], herein we investigated the in-depth chemistry of *Latrunculia biformis*, which was collected from the Antarctic Weddell Sea shelf at 291 m depth. The crude organic extract of the sponge exhibited significant in vitro anticancer activity against six cancer cell lines. A molecular networking (MN)-based metabolomics study on fractions obtained from the MeOH-soluble portion of the sponge indicated the presence of a large discorhabdin cluster with many nodes belonging to potentially new discorhabdins. Guided by anticancer activity and MN-based dereplication, six discorhabdin-type alkaloids were isolated from the MeOH subextract, including three known compounds (−)-discorhabdin L (**1**), (+)-discorhabdin A (**2**), (+)-discorhabdin Q (**3**) and three new discorhabdin derivatives, namely (−)-2-bromo-discorhabdin D (**4**), (−)-1-acetyl-discorhabdin L (**5**), and (+)-1-octacosatrienoyl-discorhabdin L (**6**). Since the amounts of the isolated compounds were very minor, only compounds **1**, **5** and **6** could be tested for their anticancer activity against the human colon cancer cell line HCT-116. We applied a structure-based docking approach on all isolated compounds and the enantiomer of **1** (**1e**) against two cancer targets reported for pyrroloiminoquinone alkaloids, i.e., topoisomerase I–II and indoleamine 2,3-dioxygenase IDO1 [19,20,21] to predict their anticancer potential and to suggest potential molecular mechanism(s) of action. This study reports MN and bioactivity-guided isolation of compounds **1**–**6**, their structure elucidation, and biological activities with potential target identification for their anticancer activity.

## 2. Results

### 2.1. Bioactivity and Molecular Networking-guided Purification and Structural Elucidation

**Figure 1:**
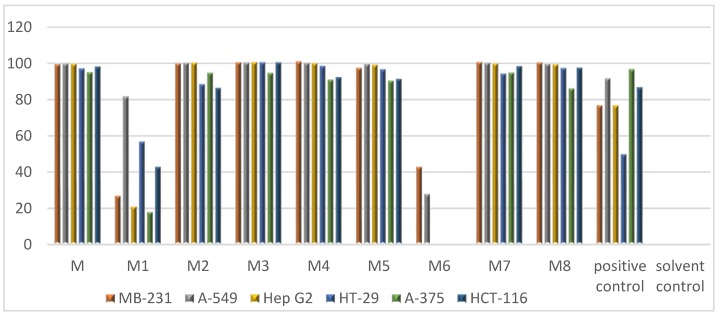
In vitro activity of MeOH subextract (M) and its C18 solid phase extraction (SPE) fractions (M1–M8) against six cancer cell lines. Test concentration: 100 µg/mL. Positive control: Doxorubicine. Solvent control: 0.5% DMSO.

The olive green-colored sponge material was freeze-dried and successively extracted with water, MeOH, and dichloromethane (DCM) subsequently. The combined organic extract was submitted to bioactivity screening against six cancer cell lines, where it showed significant activity with IC_50_ values ranging from 4.0 to 56.2 µg/mL (Table 1). The solvent partitioning of the crude organic extract between MeOH and *n*-hexane yielded the MeOH (M) and the *n*-hexane subextracts. The M subextract demonstrated strong anticancer activity (Figure 1) and was further fractionated over a C18 SPE cartridge. The anticancer activity was tracked to six SPE fractions, M2–M5, M7, and M8 (Figure 1).

In order to prioritize the isolation workflow towards undescribed molecules with potential anticancer properties, we acquired tandem UPLC-QToF-MS/MS (positive-ion mode) data on these six active fractions. The generated MS/MS (MS^2^) data were uploaded to the publicly available Global Natural Product Social Molecular Networking (GNPS) platform (http://gnps.ucsd.edu) and analyzed following the molecular networking (MN) online workflow [22]. Software Cytoscape (Version 3.61) was used to visualize the resulting networks. The automated dereplication on GNPS platform did not annotate any pyrroloiminoquinone alkaloids. Hence, compound annotation was based on manual dereplication by comparing the predicted molecular formulae against multiple public or commercially available databases. 

**Figure 2:**
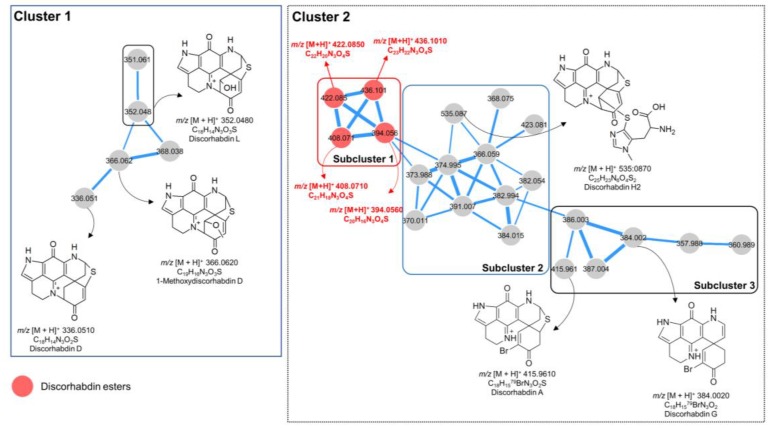
Molecular cluster observed in SPE fractions of *L. biformis* MeOH subextract. Numbers within the nodes indicate parent ions, and edge thickness represents the cosine similarity between nodes. Red nodes: Discorhabdin esters; Grey nodes: Other discorhabdin analogs.

After a comprehensive examination of the global MN of the SPE fractions, two clusters attracted our attention (Figure 2). Cluster 1 contained five nodes, four of which were annotated as known molecules discorhabdin L [13], its analog, discorhabdin D [23], and 1-methoxydiscorhabdin D [19], leaving the node at *m/z* 368.0380 to be a putatively new derivative (Figure 2). From this cluster, we were able to purify (−)-discorhabdin L (**1**), but failed to purify the potentially new discorhabdin analog (*m/z* 368.0380) due to its very minor quantity.

With 21 nodes, cluster 2 was the biggest in the generated MN, which can be further divided into three subclusters (Figure 2). Based on the elemental composition analysis, MS/MS fragmentation patterns, and biological source, two brominated alkaloids discorhabdin A [24] and discorhabdin G [25] were identified in subcluster 3. However, only discorhabdin A (**2**) was isolated in sufficient amounts for NMR and other spectroscopic analyses. Discorhabdin H2 [26] was the only annotated compound in subcluster 2. Unfortunately, neither this compound nor the remaining nodes that represent potentially new discorhabdin analogs could be purified in sufficient quantity. 

The subcluster 1 (of cluster 2) contained four nonbrominated nodes at *m/z* 394.0560, 408.0710, 422.085, and 436.1010 connected with thick edges, indicating their high structural similarity. Elemental composition analysis revealed the difference of a CH_2_ unit between these ions (Figure 2, in red). In-depth analysis of their MS/MS spectra revealed the presence of the same MS fragment (*m/z* 352.0766, C_18_H_14_N_3_O_3_S) in all four compounds, suggesting these compounds to be discorhabdin alkaloids bearing an alkyl chain with varying lengths. From subcluster 1, we isolated the compound with *m/z* 394.0560 and identified it as a new metabolite, (−)-1-acetyl-discorhabdin L (**5**), as discussed later.

In addition, we purified three compounds, namely the known compound (+)-discorhabdin Q (**3**) as well as two new compounds, namely (−)-2-bromo-discorhabdin D (**4**) and (+)-1-octacosatrienoyl-discorhabdin L (**6**), which did not appear in the MN because of the low intensity of their MS fragments. The enantiopurity of all purified compounds was further checked individually by RP-DAD-HPLC on an analytical chiral column. The sharp single peak in the UV chromatograms confirmed the enantiopurity of **1**–**6**. 

The structure of compound **1** was elucidated as (−)-(1*R*,2*S*,6*R*,8*S*)-discorhabdin L [13], based on comparison of its 1D and 2D NMR data including NOESY spectrum (Table 2 and Table 3, Supplementary Figures S1–S6). The specific rotation of compound **1** ([α]^20^_D_ = −71, *c* 0.1, MeOH) showed the same sign as that reported for (−)-discorhabdin L ([α]^20^_D_ = −240, *c* 0.0125, MeOH) [26]. In order to confirm the absolute configuration of compound **1**, the ECD (Electronic Circular Dichroism) spectrum was run. The experimental ECD spectrum of **1** (Supplementary Figure S8) was essentially identical to the ECD spectrum of (−)-(1*R*,2*S*,6*R*,8*S*)-discorhabdin L [26]. Hence, compound **1** was unambiguously characterized as (−)-(1*R*,2*S*,6*R*,8*S*)-discorhabdin L.

**Figure 3:**
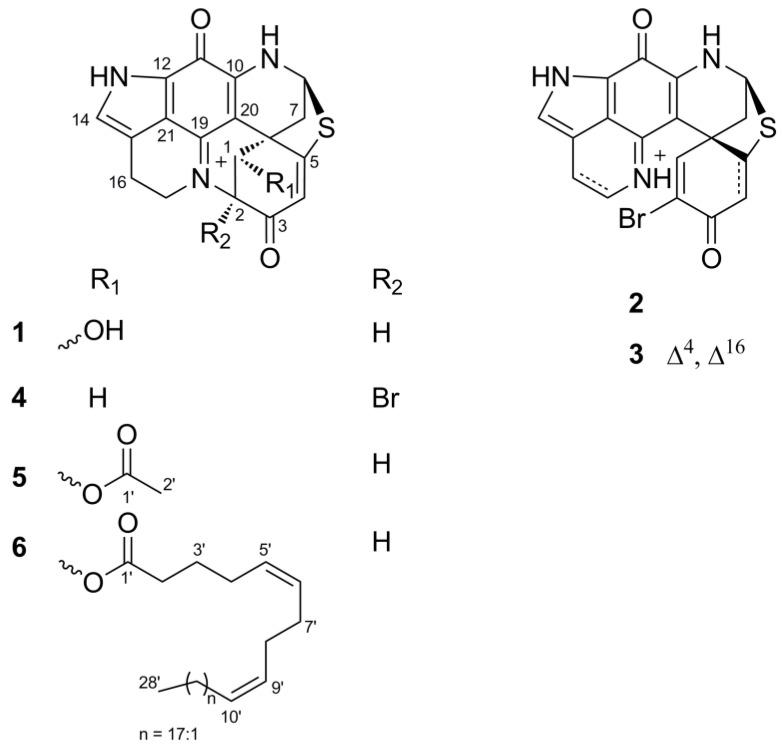
Chemical structures of compounds **1**–**6**.

Compound **2** exhibited the same ^1^H and ^13^C NMR resonances (Supplementary Figures S9 and S10) as (+)-discorhabdin A [24,27]. The analysis of its COSY, HSQC, and HMBC spectra (Supplementary Figures S10–S12) supported the same planar structure as discorhabdin A and NOESY spectrum confirmed the relative configuration of three stereocenters (Supplementary Figure S13). The specific rotation of compound **2** ([α]^20^_D_ = +197, *c* 0.01, MeOH) exhibited the same sign as (+)-discorhabdin A ([α]^20^_D_ = +400, *c* 0.05, MeOH) [24]. An early X-ray crystal analysis has confirmed the configuration of the chiral centers within (+)-discorhabdin A as 5*R*,6*S*,8*S* [27]. Thus, compound **2** was identified as (+)-(5*R*,6*S*,8*S*)-discorhabdin A (Figure 3). 

Compound **3** was identified as the known compound discorhabdin Q, based on its 1D and 2D NMR data (Supplementary Figures S15–S20), which were in good agreement with those reported in the literature [26]. The examination of the ^1^H-^1^H NOESY spectrum of **3** (Supplementary Figure S19) allowed the assignment of the relative configuration of two stereocenters. Compound **3** exhibited a specific rotation value ([α]^20^_D_ = +568, *c* 0.1, MeOH), which was similar both in the magnitude and sign to that observed for (+)-(6*S*,8*S*)-discorhabdin Q ([α]^20^_D_ = +720, *c* 0.025, MeOH) [26], hence we concluded compound **3** as (+)-(6*S*,8*S*)-discorhabdin Q.

**Figure 4:**
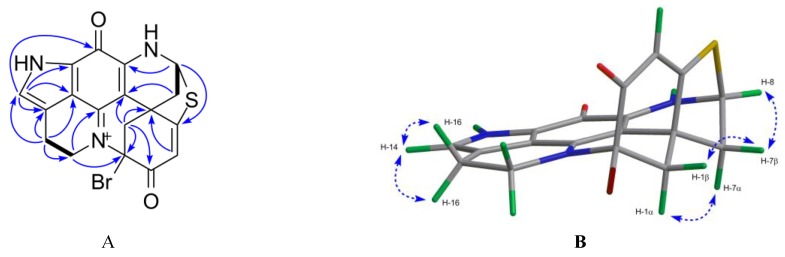
Key 2D NMR correlations observed for compound **4**. (**A**) The COSY (in bold), key H→C HMBC (arrows); (**B**) key H→H NOESY correlations (dashed line).

Compound **4** was obtained as a greenish film. The isotopic pattern of the molecule ion peaks (1:1 ratio) was indicative for the presence of one bromine atom in this molecule. The molecular formula of C_18_H_13_^79^BrN_3_O_2_S was established by the pseudo-molecular ion peak at *m/z* 413.9913 [M + H]^+^ in the HR-ESIMS (Supplementary Figure S27) spectrum, requiring 14 degrees of unsaturation. The ^1^H NMR data (Table 2, Supplementary Figure S22) together with HSQC spectrum (Supplementary Figure S23) revealed the presence of three methine resonances at *δ*_H_ 7.14 (H-14, s), *δ*_H_ 6.14 (H-4, s), and *δ*_H_ 5.68 (H-8, dd, *J* = 1.5, 3.5 Hz), four methylene groups corresponding to H_2_-17 (*δ*_H_ 3.66 and 4.62), H_2_-1 (*δ*_H_ 3.23 and 3.58), H_2_-16 (*δ*_H_ 3.10), and H_2_-7 (*δ*_H_ 2.66 and 2.84). The ^13^C NMR spectrum (Table 3) showed 18 carbon signals including 4 methylenes (*δ*_C_ 20.0, 38.7, 42.4, and 50.2), 3 methines (*δ*_C_ 63.1, 110.9, and 126.0), and 11 quaternary carbons (*δ*_C_ 44.5, 78.1, 100.4, 119.3, 122.2, 124.0, 148.2, 150.2, 165.4, 172.8, and 176.4). By comparison with the data reported for discorhabdins [13,23], the three low-field quaternary carbon signals at *δ*_C_ 176.4, 172.8, and 165.4 were tentatively assigned to C-3, C-5, and C-11, respectively, while the high-field quaternary carbon at *δ*_C_ 44.5 was assigned to C-6. The COSY correlation between H_2_-16 and H_2_-17, together with the additional ^1^H - ^13^C HMBC correlations between H-14/C-12, C-21, C-11; H_2_-16/C-14, C-21; H_2_-17/C-15, C-19 confirmed the pyrroloiminoquinone motif [13,23]. Similarly, the homonuclear COSY correlation between H_2_-7 and H-8, and the HMBC correlations between H_2_-1/C-2, C-3, C-6, C-20; H-4/C-2, C-3, C-6; and H_2_-7/C-5, C-6, C-20 suggested the position of the carbonyl group (*δ*_C_ 176.4) at C-3, and the position of the methylene (*δ*_C_ 42.4) at C-1 (Figure 4A). A further HMBC coupling between H-8 and C-5 was indicative of a thioether bridge between C-8 and C-5, while the HMBC correlation between H_2_-17 and C-2 established the bridge between N-18 and C-2 (Figure 4A). All these data, plus the lack of any further spin coupling observed for H_2_-1, allowed the placement of the bromine atom on the remaining quaternary carbon, C-2. Thus the planar structure of compound **4** was elucidated as 2-bromo-discorhabdin D. 

Compound **4** is a configurationally rigid molecule with seven rings and three stereocenters at C-2, C-6, and C-8. The relative configurations of these stereogenic centers were proposed by the NOE correlations as shown in Figure 4B. The specific rotation value of **4** ([α]^20^_D_ = −246, *c* 0.05, MeOH) is opposite to that of (+)-(2*S*,6*R*,8*S*)-discorhabdin D ([α]^20^_D_ = +80, *c* 0.025, MeOH) [26]. The experimental ECD spectrum of compound **4** (Supplementary Figure S8) showed the same cotton effects as compound **1** (−)-(1*R*,2*S*,6*R*,8*S*)-discorhabdin L. So it is reasonable to assume that compound **4** is (−)-(2*R*,6*R*,8*S*)-2-bromodiscorhabdin D. 

**Figure 5:**
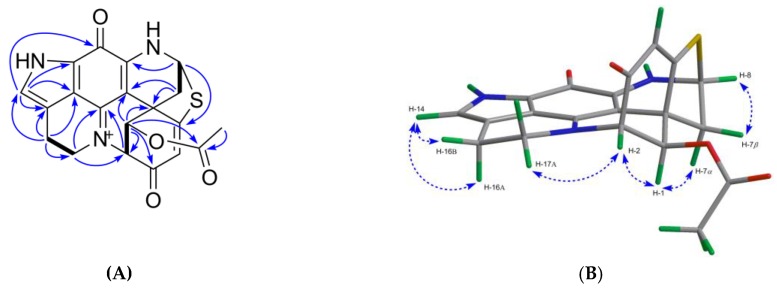
Key 2D NMR correlations observed for compound **5**. (**A**) The COSY (in bold), key H→C HMBC (arrows); (**B**) key H→H NOESY correlations (dashed line).

Compound **5** was obtained as a green film. Its molecular formula C_20_H_16_N_3_O_4_S was deduced by HR-ESIMS (*m/z* 394.0816, [M + H]^+^) indicating 15 degrees of unsaturation. The FT-IR spectrum of compound **5** displayed the characteristic ester carbonyl absorption band at *v*_max_ 1747 cm^−1^ and other similar bands as compound (**1**) at *v*_max_ 1653, 1621, 1560, 1528, 1412, and 1201 cm^−1^. The ^1^H and ^13^C NMR spectra of **5** (Table 2 and Table 3) revealed high similarity with **1**, with the only difference being the presence of an extra acetyl group in **5** (*δ*_H_ 2.15; *δ*_C_ 171.0 and *δ*_C_ 20.4). The site of esterification was identified as C-1, based on strong HMBC correlations between H-1/C-1′ and a weaker HMBC coupling between H-2′/C-1 (Figure 5A). Thus, the planar structure of compound **5** was confirmed as 1-acetyl-discorhabdin L. The analysis of the full 2D NMR dataset (COSY, HSQC, and HMBC) further confirmed that the planar structure of compound **5** (Figure 5A). The relative configuration of compound **5** was elucidated by examining its NOESY spectrum (Figure 5B). The COSY correlation between H-4/H-7 (Supplementary Figure S32) indicated a planar “W” arrangement [28] of the molecule as observed in discorhabdins L [13] and D [23], thus allowing the assignment of the resonance at *δ*_H_ 2.63 to H-7α [13]. The stereochemistry at C-1 was proposed by the strong NOE correlation (Figure 5B) between H-1/H-7α. To establish the absolute configuration of (−)-**5**, its experimental ECD spectrum was compared with that of (−)-(1*R*,2*S*,6*R*,8*S*)-discorhabdin L (**1**) (Supplementary Figure S8). The same Cotton effects observed at 290, 360, and 440 nm for both compounds established the absolute configuration of **5** as 1*R*,2*S*,6*R*,8*S*. Finally, the comparison of the specific rotation values of compounds **1** ([α]^20^_D_ = −71, *c* 0.1, MeOH) and **5** ([α]^20^_D_ = −420, *c* 0.01, MeOH) identified the structure of **5** as (−)-(1*R*,2*S*,6*R*,8*S*)-1-acetyl-discorhabdin L.

**Figure 6:**
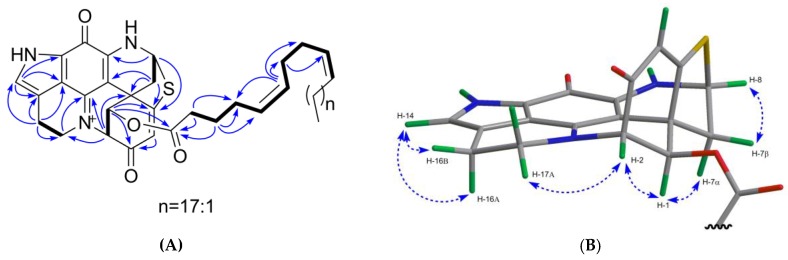
Key 2D NMR correlations observed for compound **6**. (**A**) The COSY (in bold), key H→C HMBC (arrows); (**B**) key H→H NOESY correlations (dashed line).

The most nonpolar component, compound **6,** was obtained as a green film with an [α]^20^_D_ value of + 541 (*c* 0.1, MeOH). It showed a molecular ion peak at *m/z* 752.4452 [M + H]^+^ in the HR-ESIMS spectrum. The molecular formula of C_46_H_62_N_3_O_4_S was deduced from its ^13^C NMR and HR-ESIMS data (Table 3 and Supplementary Figure S41), indicating 18 degrees of unsaturation. The FT-IR spectrum contained absorption bands typical of an ester function (*v*_max_ 1739 cm^−1^) and an alkyl chain (*v*_max_ 2927 and 2855 cm^−1^, -CH_2_ and -CH_3_ stretching bands). Comparison of the 1D-NMR data of **6** with those of **1** and **5** (Table 2 and Table 3) suggested **6** to be another analog of discorhabdin L esterified with a long chain fatty acid (*δ*_C_ 25–35 ppm; *δ*_H_ 1.2–1.4 ppm), which made this molecule very lipophilic. The discorhabdin L core structure that was evident from the 1D and 2D NMR data of **6** (Table 2 and Table 3; Figure 6) accounted for 14 degrees of unsaturation. The additional ester carbonyl at *δ*_C_ 173.6 and six sp^2^ carbons belonging to three double bonds at *δ*_C_ 129.7, 130.1, 130.8, 130.9, 131.4, and 131.7 (Supplementary Figure S36) accounted for the remaining 4 degrees of unsaturation. Hence, we concluded that the alkyl chain was an unbranched octacosa-triene-oic acid (28:3) (Table 2 and Table 3). The HMBC correlation between H-1 (*δ*_H_ 5.79) and C-1′ (*δ*_C_ 173.6) supported the attachment of the fatty acid at C-1 (Figure 6A). The geometry of these three double bonds in the fatty acid portion was elucidated by analyzing the ^13^C NMR chemical shifts of the six carbons neighboring the double bonds [29]. Carbon atoms adjacent to *cis* double bonds resonate around δ_C_ 26.0–28.5, whereas those adjacent to *trans* double bonds appear at higher chemical shift values, namely δ_C_ 29.5–38.0 [29]. The observed ^13^C NMR shifts in (+)-**6** at δ_C_ 27.5, 28.1, 28.2 (× 2), 28.4 (× 2) confirmed the *cis* (*Z*) configuration of all three double bonds in the fatty acid part (Table 3). The COSY correlations from C-2′ to C-10′ and HMBC correlations between H_2_-2′/C-1′, C-4′; H_2_-3′/C-1′, C-4′, C-5′; H-6′/C-4′, C-7′, C-8′; H_2_-7′/C-9′ (Figure 6A) allowed us to corroborate the position of two unsaturations at C-5′ and C-9′, and to assign the C-1′ to C-10′ portion of the fatty acid (Figure 3). Comparison of the 1D NMR data of (+)-**6** with the literature [30] also supports the presence of a ∆^5,9^ unsaturated fatty acid. This finding is not surprising since these *cis*,*cis*-5,9-dienoic lipids are common in sponges [31]. The chemical shift values of C-26′, C-27′ and C-28′ (Table 3) were also assigned by comparison with the literature data [32]. Thus, compound (+)-**6** was identified as C-1 octacosatrienoic acid (C28:3) ester of (−)-discorhabdin L (Figure 3). Due to availability of very minor amount of the compound (0.2 mg) and the failed attempts to improve the highly overlapped NMR signals through different solvents (e.g., MeOD, CDCl_3_, and DMSO-*d*6), we were unable to confirm the position of the third double bond. However, we believe that the lipid residue in **6** is related to the well-known 5*Z*,9*Z* demospongic acids bearing another double bond at C-17, or C-19, or C-23 [31].

On the basis of a strong NOE correlation between H-1 and H-7α (Figure 6B), as well as the other NOE correlations shown in Figure 6B, the relative configuration of **6** was elucidated to be the same as compounds **1** and **5.** The absolute configuration of **6** was established by comparing its experimental ECD spectrum with those of **1** and **5** (Supplementary Figure S8). Based on the opposite sign of the specific rotation value of **6** ([α]^20^_D_ = +541, *c* 0.1, MeOH) in comparison to compounds **1** ([α]^20^_D_ = −71, *c* 0.1, MeOH) and **5** ([α]^20^_D_ = −420, *c* 0.01, MeOH), **6** was identified as (+)-(1*R*,2*S*,6*R*,8*S*)-1-octacosatrienoyl-discorhabdin L (Figure 3).

### 2.2. In Vitro Bioactivity Tests and Molecular Docking on Purified Compounds

Anticancer activity of the pyrroloiminoquinone-type alkaloids has been the main driving force for isolation of these intriguing structural types. Due to low quantities of the isolated compounds, we were only able to assess the in vitro anticancer activities of compounds **1**, **5**, and **6** against one cell line. We used HCT-116 colon cancer cells for testing, because of the observed high activity of the MeOH subextract and SPE fractions (Table 1), plus the availability of literature data for (−)-discorhabdin L (**1**) against this cell line (IC_50_ value 6.2 µM) [17]. In the current study, compound **1** showed IC_50_ value of 0.94 µM (equal to 0.33 µg/mL). The compound **5** displayed promising activity with an IC_50_ value of 2.71 µM (= 1.1 µg/mL), while compound **6** was only modestly active against the same cell line (IC_50_ value 34.0 µM, equal to 25.6 µg/mL). These results indicate that C-1 OH function is important for anti-colon cancer activity and the substitution of the C-1 OH group, especially with a long chain fatty acyl function is not favored. 

Limited by the amounts of the isolated compounds, we performed a molecular modeling study (using Schrödinger software Maestro; www.schrodinger.com) on compounds **1**–**6** and (+)-discorhabdin L (**1e**), the (+) enantiomer of compound **1**, against two known anticancer targets (topoisomerase I/II, indoleamine 2,3-dioxygenase IDO1) to estimate their potential anticancer activity and mechanism(s) of action. Where possible, based on suitable pdb structures, docking experiments were performed. We prepared available relevant pdb protein structures, removed the original ligands, and generated receptor grids. Small molecule 3D structures of the compounds containing a quaternary nitrogen were energetically minimized and possible tautomers/protonated states were evaluated (LigPrep, counter ion not specified). Next, we docked the optimized ligand structures into respective active sites (Glide SP). Calculated 3D binding modes were illustrated, or presented as 2D ligand-interaction diagrams, for clarity.

**Figure 7:**
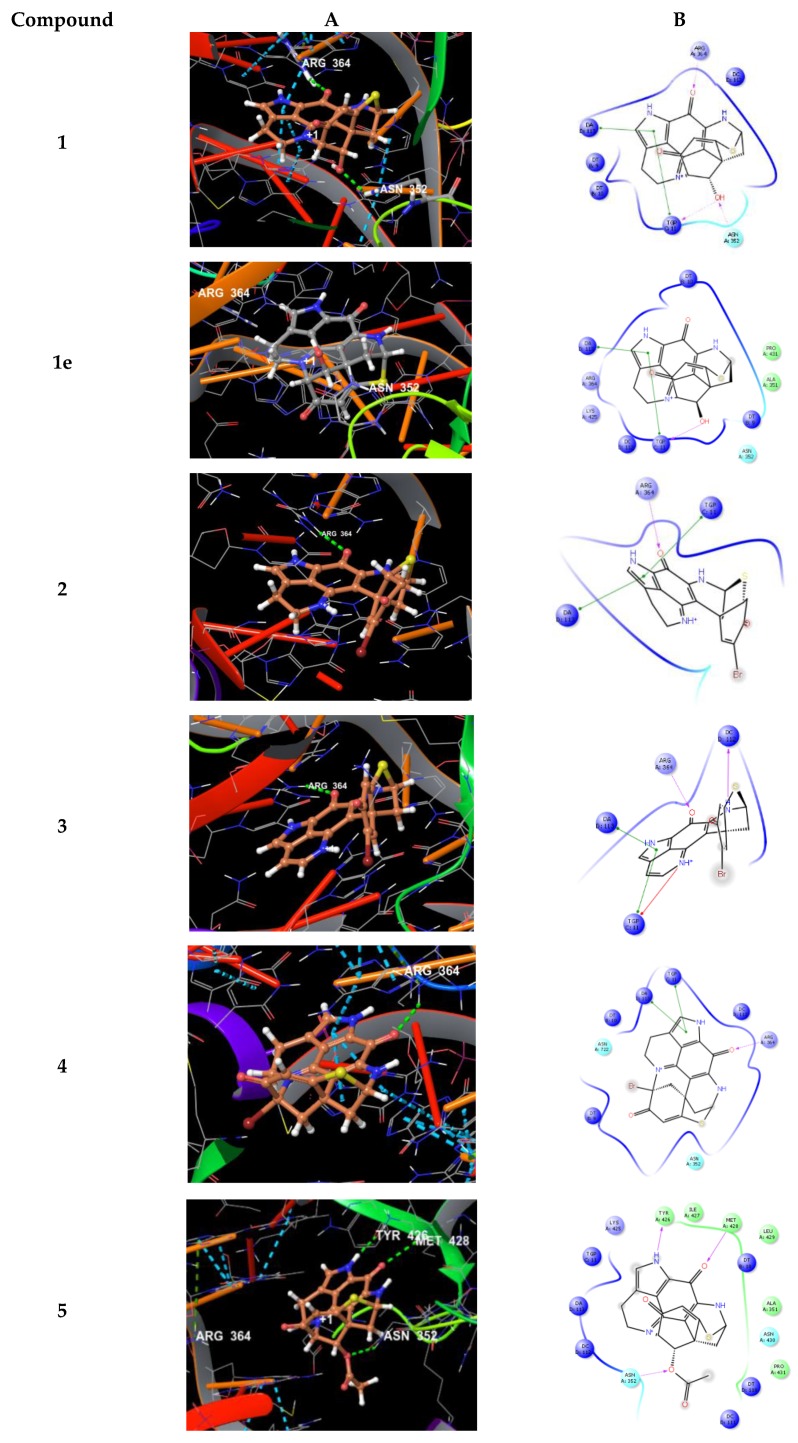
(**A**) Calculated 3D binding modes of compounds **1**–**5** and **1e** in the active site of topoisomerase I (pdb 1T8I) also containing a DNA molecule (colored in red) with a single strand break; (**B**) corresponding 2D ligand interaction diagrams showing key interactions of compounds **1**–**5** and **1e** towards topoisomerase I and DNA.

Docking of compounds **1**–**5** into the active site of topoisomerase I (pdb 1T8I) yielded plausible binding modes (Figure 7), while no binding pose could be calculated for compound **6**, due to the sterically demanding side chain that did not fit into the tight binding pocket). Compared to the original ligand camptothecin, the flat, partly aromatic core of the discorhabdins **1**–**5** intercalated into the DNA part thereby forming aromatic π-π-stacking interactions while also addressing H-bonds towards residues of the topoisomerase I protein. Similar results were obtained by docking experiments with topoisomerase II (pdb 3QX3, original ligand etoposide) suggesting these proteins to be anticancer targets for the compounds **1**–**5**. Molecular docking study performed (in analogy to the procedure described above) on compound **1e**, which was reported to exhibit strong in vitro cytotoxicity [33], revealed a binding mode in the active site of topoisomerase I, too (Figure 7). Comparable to **1**, the flat core forms a DNA-intercalating complex, but the ligand is distorted by 180°. Thus, the binding modes of both compounds, **1** and **1e**, suggest those ligands to be rather non-specific DNA intercalators.

**Figure 8:**
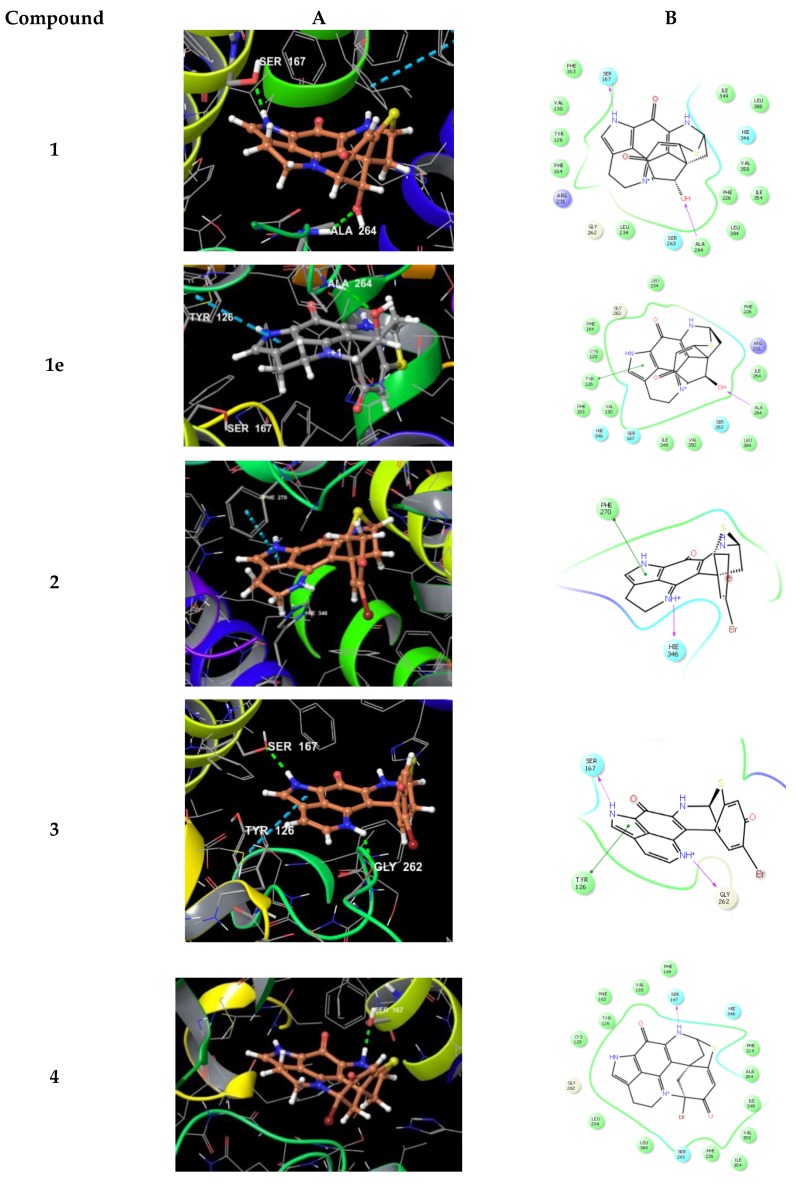
3D binding poses (**A**) and ligand interaction diagrams (**B**) of compounds **1**–**4** and **1e** in the active site of IDO1 (pdb 6AZW). Key interactions are shown. The binding pocket is shown in a similar orientation, respectively. Ligand docking revealed plausible binding poses for compounds **1**–**4** and **1e**, but not for compounds **5** and **6**.

We also performed docking experiments with another reported target for pyrroloiminoquinone alkaloids, namely the indoleamine 2,3-dioxygenase (IDO1) enzyme for which structural data including ligand–protein complexes are available. As cofactor to mediate physiological substrate oxidation, IDO1 contains a heme moiety and ligands typically form interactions by complexing the iron central atom. Examples for such IDO1 inhibitors and relevant interacting moieties include NLG919 derivative (imidazole-like nitrogen in pdb 5EK2) or ligand INCB14943 (hydroxylamidine moiety in pdb 5XE1). Since the compounds lack comparable nitrogen functionalities to able to interact with heme in a similar manner, docking of the compounds into these active sites revealed no plausible binding modes. However, recent reports demonstrated that another class of potent IDO1 inhibitors such as FXB-001116 (pdb 6AZW) and BMS-978587 (pdb 6AZV) bind to the IDO1 apo structure with high affinity, thereby displacing the heme moiety. Accordingly, we performed docking experiments of compounds **1**–**6** and **1e** using the apo-protein. This approach suggested possible binding modes for compounds **1**–**4** and **1e** in the apo active site of IDO1 (Figure 8), but not for the sterically more demanding compounds **5** and **6**.

Inspecting the docking poses of **1** in the above-mentioned protein structures on a molecular level pointed towards a key role for the sterically defined OH-function in **1**, as it mediates an H-bond towards Ala264. Another key H-bond interaction occurred between aromatic NH of **1** towards Ser167 (Figure 8). Compound **1** shows a rather planar core, anchoring the ligand with a strong shape-fit into the tight binding pocket. Relative to this core, the thioether bridge sits almost rectangular on top, occupying a lipophilic area within the binding site, flanked by residues including Val350, Phe226, and Leu384. Within these small sets of compounds, docking of **1** reveals the optimal pose. We also docked (+)-discorhabdin L (**1e**), the enantiomer of **1,** into the active site of IDO1 (in analogy to the procedure described above). Interestingly, the flat core was found to again fill the rather flat pocket. In comparison to **1**, the core sits upside down in **1e**, with the key H-bond towards Ala264 being maintained. Accordingly, the NH-bond towards Ser167 was lost, but the aromatic system formed a π-interaction to Tyr126 (Figure 8). These poses suggest the flat aromatic core of this type of compounds to be the main requisite to bind into the pocket. Furthermore, stereochemistry seems to play a minor role in binding. Thus, it can be assumed the compounds are rather non-specific IDO1-ligands.

The calculated binding mode of compound **4** yielded a shifted orientation of the core, with only one H-bond towards Ser167 (Figure 8). In contrast, compounds **5** and **6** gave no plausible docking solutions, again due to the sterically demanding ester moieties, thus also preventing H-bonding by OH towards Ala264. 

In summary, the molecular modeling data suggested plausible binding modes for compounds **1**–**5** in the target structure of topoisomerase I, and for compounds **1**–**4** towards IDO1, respectively. However, it is well possible that this class of compounds bind further key anticancer targets, contributing to their cytotoxic potential.

## 3. Discussion

Since the discovery of discorhabdin C from a New Zealand *Latrunculia* sp. in 1986 [8], more than 40 discorhabdin analogs have been reported from different marine sponge genera [34]. Some discorhabdins contain bromination at C-2, C-4, or C-14 positions (e.g., discorhabdin A, discorhabdin C, 14-bromodihydrodiscorhabdin C) [8,23,24,35], some possess a sulfur bridge between C-5 and C-8 (e.g., discorhabdin B, discorhabdin Q) [24,36]. A few discorhabdins (e.g., discorhabdin L, discorhabdin D) are heptacyclic through the formation of an extra bridge between C-2 and N-18 [13,24]. Of the six compounds obtained in the current study, two of them (**5** and **6**) are (−)-discorhabdin L esters. Compound **6** is a triunsaturated C28 fatty acid ester of discorhabdin L. To our knowledge this is the first discorhabdin alkyl ester structure being reported from a marine sponge. Notably, Zou et al. (2013) reported atkamine, a new, large pyrroloiminoquinone scaffold containing a fused epoxybenzazepin and bromophenol groups connected with a cyclic sulfide ring [37]. Between the former and the latter rings, there is a substitution with a monosaturated C20 alkyl chain. The authors suggested this alkyl group to originate from (*Z*)-15-docosenoic acid, a fatty acid commonly found in sponge species that was possibly incorporated in the very early biosynthesis stages of atkamine [37]. Compounds **5** and **6** isolated in this study instead bear an esterification at C-1 position of the pyrroloiminoquinone ring system. Compound **5** is an acetyl ester of (−)-discorhabdin L**,** while **6** is an ester of discorhabdin L with an unbranched octacosatrienoic acid. Marine sponges, especially demosponges, are regarded as one of the richest sources of long-chain fatty acids (LCFAs; i.e., C23–34) [38,39]. The octacosatrienoic acid (C28:3) has been reported from marine sponges and corals [40,41,42], but not from any *Latrunculia* species. The only study that analyzed the FAs composition of Antarctic *Latrunculia* sponges in 2015 showed that Antarctic *L. biformis* contained diverse common and LCFAs (C16, C18), however longer chain unsaturated FAs were not found [43]. This is the first report of a discorhabdin-LCFA ester from nature, and the current study adds three new and intriguing analogs to the list of discorhabdin class alkaloids.

Discorhabdins have been repeatedly studied for their in vitro anticancer activity [10,13,14]. Limited by the strong cytotoxicity and the supply issue, no molecule from this chemical family has ever proceeded to further clinical studies. A few structure–activity relationship studies (SARs) associated with discorhabdins have been conducted, revealing that the ring closure by a bridge between C2 and N18 can significantly reduce the cytotoxicity, while a substitution at C-1 (i.e., OMe, NH_2_) can enhance the anticancer activity [19,26]. The discovery of C-1 esters of discorhabdin L with good to moderate inhibitory activity against HCT-116 cell line here provides further insights for the SARs of discorhabdins. 

Although many discorhabdins are associated with anticancer/cytotoxic activity, little is known on their exact mechanism(s) of action. Wada et al. (2011) evaluated (+)-discorhabdin A and its synthetic oxa analog for inhibition against a set of anticancer target enzymes, such as protein kinase, histone deacetylase, farnesyltransferase, telomerase, and proteasome [16]. (+)-Discorhabdin A and its synthetic oxa analog weakly inhibited the farnesyltransferase enzyme (IC_50_ > 10µM) [16]. Geoy et al. recently tested the HIF-1α/p300 inhibition activity of several discorhabdins, including (−)-discorhabdin L (IC_50_ value 0.73 μM) concluding them to be a novel class of HIF-1α/p300 inhibitors [17]. In the current study, an *in silico* molecular modeling study revealed the plausible binding modes of discorhabdins in two additional cancer target enzymes, topoisomerase I/II and IDO1.

In summary, guided by the anticancer activity and MN-based metabolomics, the Antarctic deep-sea sponge *L. biformis* led to the isolation and characterization of three known and three new discorhabdin alkaloids. Despite the small amounts of the extract and fractions that hampered the isolation of many further new discorhabdins, MN-based metabolomics proved useful for identification of chemical inventory of the sponge in early stages. Two compounds were a new type of discorhabdin esters that yielded meaningful SARs in comparison to the parent compound discorhabdin L. Mechanistic studies based on molecular modeling showed, for the first time, the potential binding of discorhabdins to additional anticancer targets that may be involved in their anticancer activity. 

## 4. Materials and Methods 

### 4.1. General Procedures 

Specific rotation of compounds **1**–**6** were measured on a Jasco P-2000 polarimeter (Jasco, Pfungstadt, Germany). FT-IR spectra were recorded using a PerkinElmer Spectrum Two FT-IR spectrometer (PerkinElmer, Boston, MA, USA). UV spectra were run on a NanoVue Plus spectrophotometer (GE Healthcare, New York, NY. USA). ECD spectra were run in MeOH on a J-810 CD spectrometer (Jasco, Pfungstadt, Germany). NMR spectra were obtained on a Bruker AV 600 spectrometer (600 and 150 MHz for ^1^H and ^13^C NMR, respectively, Bruker^®^, Billerica, MA, USA) equipped with 5.0 mm Shigemi tube (SHIGEMI, Co., LTD., Tokyo, Japan). The residual solvent signals were used as internal references: *δ*_H_ 3.31/*δ*_C_ 49.0 ppm (MeOD), and *δ*_H_ 2.50/*δ*_C_ 39.51 ppm (DMSO-*d_6_*). 4-Dimethyl-4-silapentane-1-sulfonic acid (DSS) served as the internal standard. HRMS/MS data were recorded on a Waters Xevo G2-XS QTof Mass Spectrometer (Waters^®^, Milford, MA, USA) coupled to a Waters Acquity I-Class UPLC system (Waters^®^, Milford, MA, USA). HR-ESIMS was recorded on micrOTOF II-High-performance TOF-MS system (Bruker^®^, Billerica, MA, USA) equipped with an electrospray ionization source. Solid phase extraction (SPE) was performed on the Chromabond SPE C18 column cartridges (6 mL/2000 mg, Macherey-Nagel, Duren, Germany). HPLC separations were performed on a VWR Hitachi Chromaster system (VWR International, Allison Park, PA, USA) consisting of a 5430-diode array detector (VWR International, Allison Park, PA, USA), a 5310-column oven, a 5260 autosampler, and a 5110 pump combined in parallel with a VWR evaporative light scattering detector (ELSD 90, VWR International, Allison Park, PA, USA). The eluents used for HPLC separations were H_2_O (A) and MeCN (B). Routine HPLC separations were performed on a semi-preparative C18 monolithic column (Onyx, 100 × 10 mm, Phenomenex, Torrance, CA, USA) and an analytical synergi column (250 × 4.6 mm, Phenomenex, Torrance, CA, USA). A chiral cellulose-1 column (Lux 5µ, 250 × 4.6 mm, Phenomenex, Torrance, CA, USA) was used for checking the enantiopurity of each purified compound. The organic solvents used for UPLC-QToF-MS/MS analyses were ULC/MS grade (Biosolve BV, North Brabant, Netherlands) and HPLC grade (ITW Reagents, Darmstadt, Germany) for HPLC isolation processes. The water used was MilliQ-water produced by Arium^®^ Water Purification Systems (Sartorius, Göttingen, Germany).

### 4.2. Sponge Material

The sponge was collected in 2015/2016 during the Expedition PS96 of the Research Vessel POLARSTERN to the southern Weddell Sea (Antarctica). The sponge was collected by an Agassiz trawl at a depth of −291 m, and was fixated immediately after collection. Specimens were cleaned, pre-sorted, photographed, and transferred into buckets with cold seawater as soon as the catch was on deck. Subsamples were transferred into pure ethanol (96%) and the main parts were frozen at −20 °C. The sponges were transported to the Senckenberg Research Institute and Nature Museum in Frankfurt am Main, Germany. Tissue samples were taken and skeletal preparations were made for transmission light microscopy and SEM, according to standard protocols [44]. For taxonomic examination, the sponge spicules were mounted on microscope slides and studied by light microscopy and by SEM. Based on comparative morphology of skeletal characters, the sponge was identified as *Latrunculia biformis*, which is a common species in the Antarctic deeper shelf areas. For identification, the World Porifera Database [45] and relevant literature were used. A specimen (SMF 12109) is deposited in the Porifera collection of Senckenberg Research Institute and Nature Museum, electronically inventoried. The data are online available in the SESAM database.

### 4.3. Extraction and Isolation 

The sponge material (43.615 g, frozen weight) was cut into small pieces and freeze-dried (Martin Christ, Osterode am Harz, Germany). The lyophilized biomass (5.809 g) was extracted at room temperature with water (3 × 200 mL) under agitation to yield the aqueous extract (1.892 g). The remaining sponge residue (3.572 g, dry weight) was extracted with MeOH (3 × 150 mL) and subsequently with DCM (3 × 150 mL) under the same conditions. Combined MeOH and DCM extracts were evaporated to dryness by a rotary evaporator to yield the crude organic extract (328 mg) that showed very strong anticancer activity against multiple cancer cell lines. This extract was partitioned between MeOH (100 mL) and *n*-hexane (100 mL) to yield MeOH (190 mg) and *n*-hexane (120 mg) subextracts. The MeOH-soluble portion, which exhibited strong anticancer activity was fractionated on a Chromabond SPE C18 cartridge. The elution with a step gradient MeOH:H_2_O mixture (0% to 100%) afforded 8 fractions (M1–M8), of which the anticancer activity was tracked to six fractions M2–M5, M7, and M8. RP-HPLC separation of M2 (18 mg) on the analytical Synergi column gradient of H_2_O:MeCN (77:22), with 0.1% TFA, flow 1.0 mL/min yielded compound **1** (1.5 mg, *t*_R_ 5.5 min). RP-HPLC analysis of M3 (10 mg) on the same column (gradient of H_2_O:MeCN from 80:20 to 70:30 in 25 min, with 0.1% TFA, flow 1.0 mL/min) afforded compounds **5** (0.3 mg, *t*_R_ 15.2 min) and **3** (0.3 mg, *t*_R_ 19.0 min). M4 (16 mg) was further fractionated on a Chromabond SPE C18 cartridge to furnish 5 subfractions (M4-1 to M4-5). The subfraction M4-1 (4.4 mg) was further purified by RP-HPLC equipped with an analytical C18 column using a gradient of H_2_O:MeCN (87:13 to 80:20, 0–16 min, 80:20 to 74:26, 16–27 min, with 0.1% TFA, flow 1.0 mL/min) to yield compounds **4** (0.1 mg, *t*_R_ 18.9 min) and **2** (0.1 mg, *t*_R_ 20.5 min). RP-HPLC separation of the nonpolar fraction M8 (23.9 mg) (gradient of H_2_O:MeCN 25:75 to 0:100, 0–15 min, with 0.1% TFA, flow 1.0 mL/min) on an analytical C18 column yielded compound **6** (0.2 mg, *t*_R_ 14.9 min). Each purified compound was further checked, individually, for enantiopurity by RP-DAD-HPLC on a chiral analytical column using a gradient of H_2_O:MeCN (99:1 to 0:100, 0–15 min, with 0.1% TFA, flow 1.5 mL/min). 

*(*−*)-(1R,2S,6R,8S)-Discorhabdin L* (**1**): Greenish film; [α]^20^_D_ = −71 (*c* 0.1, MeOH); ^1^H NMR (CD_3_OD, 600 MHz) and ^13^C NMR (CD_3_OD, 150 MHz) Table 2 and Table 3; HR-ESIMS found *m/z* [M + H]^+^ 352.0748, C_18_H_14_N_3_O_3_S requires 352.0756. 

*(+)-(5R,6S,8S)-Discorhabdin A* (**2**): Orange film; [α]^20^_D_ = +197 (*c* 0.01, MeOH); HR-ESIMS found *m/z* [M + H]^+^ 416.0065, C_18_H_15_^79^BrN_3_O_2_S requires 416.0068.

*(+)-(6S,8S)-Discorhabdin Q* (**3**): Orange film; [α]^20^_D_ = +568 (*c* 0.1, MeOH); HR-ESIMS found *m/z* [M + H]^+^ 411.9733, C_18_H_11_^79^BrN_3_O_2_S requires 411.9759. 

*(*−*)-(2R,6R,8S)-2-Bromodiscorhabdin D* (**4**): Greenish film; UV (MeOH) *λ*_max_ 250 (*є* 13840), 285 (*є* 11184), 325 (*є* 7947), 403 (*є* 7802) nm; [α]^20^_D_ = −246 (*c* 0.05, MeOH); IR (film) *v*_max_ 2922, 2852, 1657, 1533, 1514, 1432, 1230 cm^−1^; ^1^H NMR (CD_3_OD, 600 MHz) and ^13^C NMR (CD_3_OD 150 MHz) Table 2 and Table 3; HR-ESIMS found *m/z* [M + H]^+^ 413.9913, C_18_H_13_^79^BrN_3_O_2_S requires 413.9912. 

*(*−*)-(1R,2S,6R,8S)-1-Acetyl-discorhabdin L* (**5**): Greenish film; UV (MeOH) *λ*_max_ 250 (*є* 24822), 283 (*є* 17019), 325 (*є* 11700), 403 (*є* 11169) nm; [α]^20^_D_ = −420 (*c* 0.01, MeOH); IR (film) *v*_max_ 2926, 2854, 1747, 1653, 1621, 1560, 1528, 1412, 1201 cm^−1^; ^1^H NMR (CD_3_OD, 600 MHz) and ^13^C NMR (CD_3_OD, 150 MHz) Table 2 and Table 3; HR-ESIMS found *m/z* [M + H]^+^ 394.0816, C_20_H_16_N_3_O_4_S requires 394.0861. 

*(+)*-*(1R,2S,6R,8S)-1-Octacosatrienoyl-discorhabdin L* (**6**): Greenish film; UV (MeOH) *λ*_max_ 203 (*є* 19890), 249 (*є* 19439), 285 (*є* 15904), 325 (*є* 13122), 403 (*є* 11543) nm; [α]^20^_D_ = +541 (*c* 0.1, MeOH); IR (film) *v*_max_ 3007, 2927, 2855, 1739, 1678, 1621, 1566, 1527, 1441, 1206, 1185, 1135 cm^−1^; ^1^H NMR (CD_3_OD, 600 MHz) and ^13^C NMR (CD_3_OD, 150 MHz) Table 2 and Table 3; HR-ESIMS found *m/z* [M + H]^+^ 752.4452, C_46_H_62_N_3_O_4_S requires 752.4461.

### 4.4. UPLC-QToF-MS/MS Analysis

The six active C18 SPE fractions of the MeOH soluble portion were analyzed on an ACQUITY UPLC I-Class System coupled to the Xevo G2-XS QToF Mass Spectrometer (Waters^®^, Milford, Massachusetts, USA) equipped with an electrospray ionization (ESI) source operating with a positive polarity at a mass range of *m/z* 50–1600 Da. The 0.1 mg/mL MeOH solution of the fractions were filtered through a 0.2 μm PTFE syringe filter (Carl Roth, Karlsruhe, Germany) and then injected (injection volume: 1.0 μL) into the system equipped with Acquity UPLC HSS T3 column (high-strength silica C18, 1.8 µm, 100 × 2.1 mm I.D., Waters^®^) operating at 40 °C. Separation was achieved with a binary LC solvent system controlled by MassLynx^®^ (version 4.1) using mobile phase A 99.9% water/0.1% formic acid (ULC/MS grade) and B 99.9% ACN/0.1% formic acid (ULC/MS grade), pumped at a rate of 0.6 mL/min with the following gradient: Initial, 1% B; 0.0–12.0 min to 100% B; 12.0–13.0 min 100% B, and a column reconditioning phase until 15 min. 

ESI conditions were set with the capillary voltage at 0.8 kV, sample cone voltage at 40.0 V, source temperature at 150 °C, desolvation temperature at 550 °C, cone gas flow in 50 L/h, and desolvation gas flow in 1200 L/h. MS/MS setting was linear collision energy (CE) at 30 eV. As a control, solvent (methanol) was injected. MassLynx^®^ (Waters^®^, V4.1) was used to analyze the achieved MS and MS^2^ data.

### 4.5. Molecular Networking

The network was created using the UPLC-HRMS/MS data generated from the six active MeOH subfractions of *L. biformis*. All raw MS/MS data were converted from files (.raw) to mzXML file format using MSConvert (Version 3.6.10051, Vanderbilt University, Nashville, TN, USA). The converted data files were uploaded to the Global Natural Products Social molecular networking (http://gnps.ucsd.edu) platform using FileZilla (https://filezilla-project.org/) and a molecular network was created using the online workflow at GNPS [22]. The data were filtered by removing all MS/MS peaks within +/− 17 Da of the precursor *m/z*. MS/MS spectra were window filtered by choosing only the top 6 peaks in the +/− 50Da window throughout the spectrum. The data were then clustered with MS-Cluster with a parent mass tolerance of 0.1 Da and an MS/MS fragment ion tolerance of 0.05 Da to create consensus spectra. Further, concensus spectra that contained less than 2 spectra were discarded. A network was then created where edges were filtered to have a cosine score above 0.6 and more than 3 matched peaks. Further edges between two nodes were kept in the network if and only if each of the nodes appeared in each other’s respective top 10 most similar nodes. The spectra in the network were then searched against GNPS’ spectral libraries. The library spectra were filtered in the same manner as the input data. All matches kept between network spectra and library spectra were required to have a score above 0.7 and at least 6 matched peaks. The output molecular networking data were analyzed and visualized using Cytoscape (ver. 3.61) [46]. 

### 4.6. Cytotoxicity Assay

Crude extract of *L. biformis* and downstream fractions were tested in vitro at a final concentration of 100 µg/mL against 6 human cancer cell lines, Hep G2 (liver cancer cell line, DSMZ, Braunschweig, Germany), HT29 (colorectal adenocarcinoma cell line, DSMZ, Braunschweig, Germany), A375 (malignant melanoma cell line, CLS, Eppelheim, Germany), HCT116 (colon cancer cell line, DSMZ, Braunschweig, Germany), A549 (lung carcinoma cell line, CLS, Eppelheim, Germany), and MDA-MB231 (human breast cancer line, CLS, Eppelheim, Germany). Cells were supplemented at 37 °C and 5% CO_2_ in RPMI 1640 medium (Life Technologies, Darmstadt, Germany) with 10% fetal bovine serum, 100 U/mL penicillin and 100 mg/mL streptomycin. A stock solution of 20 mg/mL in DMSO was prepared for each test sample. After 24 h incubation in 96-well plates, the medium in the cells was replaced by 100 µL fresh medium containing the test samples and cells were incubated for another 24 h at 37 °C. Doxorubicin was used as positive control, while 0.5% DMSO and growth media served as negative controls. All samples were prepared in duplicates. The assay was performed according to the manufacturer’s instructions (Promega, Madison, WI, USA). Cells were incubated for 2 h at 37 °C and fluorescence at an excitation wavelength of 560 nm and emission at 590 nm was measured. For the determination of IC_50_ values, a dilution series of the extracts were tested following the same procedure as described before. IC_50_ values were calculated by using Excel to determine the concentration that shows 50% inhibition of the viability.

### 4.7. Molecular Modeling and Docking

Molecular modeling was performed on a DELL Precision T3610 four core workstation using Schrödinger Maestro (version 11.3, 2017, Schrödinger, LLC, New York, NY, USA). The following RCSB protein data bank (pdb) crystal structures were used for modeling studies: 1T8I, 3QX3, 5EK2, 5XE1, 6AZV, 6AZW. Each protein structure was initially prepared by standard settings of the Protein Preparation Wizard 2015-4 (Epik version 2.4, Schrödinger, LLC, 2015; Impact version 5.9, Schrödinger, LLC, 2015; Prime version 3.2, Schrödinger LLC, 2015). For energy minimizations of the small-molecule ligands, MacroModel (version 11.0, Schrödinger, LLC, 2015) was used. Ionization states and tautomers were generated with LigPrep (version 3.6, Schrödinger, LLC, 2015). Ligand docking and receptor grid generation was performed with Glide (version 6.9, Schrödinger, LLC, 2015). Figures and ligand interaction diagrams (LID) were generated by Maestro.

## Figures and Tables

**Table 1:** Anticancer activity of the *L. biformis* crude extract. The IC_50_ values are in µg/mL. Positive control doxorubicine.

Sample	A-375	HCT-116	A-549	MB-231	Hep G2	HT-29
Crude extract	17.4	4.8	56.2	46.8	18.2	4.0
Positive control	0.13	10.6	31.4	15.2	14.6	3.0

**Table 2:** ^1^H NMR data of compounds **1**, **4, 5**, and **6** in CD_3_OD (trifluoroacetic acid (TFA) salts, 600 MHz, *δ* in ppm).

NO.	1	4	5	6
	*δ*_H_, Mult. (*J* in Hz)	*δ*_H_, Mult. (*J* in Hz)	*δ*_H_, Mult. (*J* in Hz)	*δ*_H_, Mult. (*J* in Hz)
1	4.63 d (3.6)	3.58 d (13.3) 3.23 d (13.3)	5.79 d (3.6)	5.79 d (3.6)
2	4.15 d (3.6)	-	4.36 d (3.6)	4.35 d (3.6)
4	6.14 s	6.14 s	6.23 s	6.23 s
7α	2.57 dd (1.3, 12.0)	2.66 dd (1.5, 12.1)	2.63 dd (1.4, 12.1)	2.64 d (1.2, 12.1)
7β	2.96 dd (3.6, 12.0)	2.84 dd (3.5, 12.1)	2.81 dd (3.7, 12.1)	2.76 dd (3.6, 12.1)
8	5.59 dd (1.3, 3.6)	5.68 dd (1.5, 3.5)	5.61 dd (1.4, 3.7)	5.61 dd (1.2, 3.6)
14	7.11 s	7.14 s	7.13 s	7.13 s
16	3.19 ddd (7.5, 13.0, 16.7) 3.06 ddd (3.0, 6.9, 16.7)	3.10 m	3.21 ddd (6.9, 7.5, 16.6) 3.08 ddd (2.9, 6.9, 16.6)	3.22 ddd (6.8, 7.3, 16.6) 3.08 ddd (2.7, 6.8, 16.6)
17	4.02 ddd (3.0, 7.5, 14.2)	4.62 ddd (2.1, 5.6, 13.8)	4.04 ddd (2.9, 7.5, 13.8)	4.04 ddd (2.7, 7.3, 13.7)
	3.91 ddd (6.9, 13.0, 14.2)	3.66 td (6.3, 13.8)	3.93 td (6.9, 13.8)	3.93 td (6.8, 13.7)
2′	-	-	2.15 s	2.44 td (1.5, 7.5)
3′	-	-	-	1.69 m
4′	-	-	-	2.10 m
5′	-	-	-	5.34 m
6′	-	-	-	5.44 m
7′	-	-	-	2.08 m
8′	-	-	-	2.08 m
9′	-	-	-	5.37 m
10′	-	-	-	5.34 m
11′–27′	-	-	-	1.25–1.40 m; 2.00–2.06 m; 5.36 m
28′	-	-	-	0.90 t (7.0)

**Table 3:** ^13^C NMR data of compounds **1**, **4, 5**, and **6** in CD_3_OD (150 MHz, *δ* in ppm).

Position	1	4	5	6
	*δ*_C_	*δ*_C_^a^	*δ*_C_	*δ*_C_
1	68.5 (CH)	42.4 (CH_2_)	69.6 (CH)	69.5 (CH)
2	67.8 (CH)	78.1 (C)	64.6 (CH)	64.6 (CH)
3	184.8 (C)	176.4 (C)	183.1 (C)	183.0 (C)
4	114.1 (CH)	110.9 (CH)	114.4 (CH)	114.4 (CH)
5	171.5 (C)	172.8 (C)	171.2 (C)	171.1 (C)
6	48.6 (C)	44.5 (C)	47.0 (C)	47.1 (C)
7	37.4 (CH_2_)	38.7 (CH_2_)	37.4 (CH_2_)	37.5 (CH_2_)
8	63.7 (CH)	63.1 (CH)	63.5 (CH)	63.5 (CH)
10	148.6 (C)	148.2 (C)	149.0 (C)	149.1 (C)
11	167.5 (C)	165.4 (C)	167.1 (C)	167.1 (C)
12	125.6 (C)	124.0 (C)	125.6 (C)	125.6 (C)
14	127.2 (CH)	126.0 (CH)	127.4 (CH)	127.4 (CH)
15	119.2 (C)	119.3 (C)	119.4 (C)	119.4 (C)
16	20.6 (CH_2_)	20.0 (CH_2_)	20.7 (CH_2_)	20.7 (CH_2_)
17	52.8 (CH_2_)	50.2 (CH_2_)	52.9 (CH_2_)	52.9 (CH_2_)
19	150.3 (C)	150.2 (C)	150.4 (C)	150.4 (C)
20	101.8 (C)	100.4 (C)	100.6 (C)	100.6 (C)
21	122.7 (C)	122.2 (C)	122.7 (C)	122.7 (C)
1′	-		171.0 (C)	173.6 (C)
2′	-		20.4 (CH_3_)	34.0 (CH_2_)
3′	-		-	25.8 (CH_2_)
4′	-		-	27.5 (CH_2_)
5′	-		-	129.7 (CH)
6′	-		-	131.7 (CH)
7′	-		-	28.4 (CH_2_)
8′	-		-	28.4 (CH_2_)
9′	-		-	130.1 (CH)
10′	-		-	130.8 (CH)
11′–25′	-		-	28.1–30.9 (CH_2_); 130.9 (CH); 131.4 (CH)
26′	-		-	32.9 (CH_2_)
27′	-		-	23.7 (CH_2_)
28′	-		-	14.5 (CH_3_)

^a^ Extracted from HSQC and HMBC spectra.

## Supplementary Materials

The following are available online at https://www.mdpi.com/1660-3397/17/8/439/s1, HR-ESIMS and NMR spectra of compounds **1**–**6**. ECD spectra of compounds **1**,**4**,**5**, and **6**.
