# Supplementary material for: New Discorhabdin Alkaloids from the Antarctic Deep-Sea Sponge Latrunculia biformis
Source: Mar Drugs. 2019 Jul 25;17(8):439. doi: 10.3390/md17080439 (PMC6722921; doi:10.3390/md17080439)

SUPPLEMENTARY MATERIALS

New Discorhabdins from the Antarctic Deep-Sea Sponge *Latrunculia biformis*

Fengjie Li [1], Christian Peifer [2], Dorte Janussen [3], and Deniz Tasdemir [1,4*]

[1] GEOMAR Centre for Marine Biotechnology (GEOMAR-Biotech), Research Unit Marine Natural Products Chemistry, GEOMAR Helmholtz Centre for Ocean Research Kiel, Am Kiel-Kanal 44, 24106, Kiel, Germany; fli@geomar.de

[2] Pharmaceutical Chemistry, Kiel University, Gutenbergstraße 76, 24118 Kiel, Germany; cpeifer@pharmazie.uni-kiel.de

[3] Senckenberg Research Institute and Natural History Museum, Senckenberganlage 25, D-60325 Frankfurt, Germany; dorte.janussen@senckenberg.de

[4] Faculty of Mathematics and Natural Sciences, Kiel University, Christian-Albrechts-Platz 4, 24118 Kiel, Germany;

* Correspondence: dtasdemir@geomar.de; Tel.: +49-431-600-4430

Figure No

Figure S1. ^{1}H NMR spectrum of compound **1** (TFA salt, 600 MHz, CD_3OD).

Figure S2. ^{13}C NMR spectrum of compound **1** (TFA salt, 150 MHz, CD_3OD).

Figure S3. HSQC spectrum of compound **1** (TFA salt, 600 MHz, CD_3OD).

Figure S4. HMBC spectrum of compound **1** (TFA salt, 600 MHz, CD_3OD).

Figure S5. COSY spectrum of compound **1** (TFA salt, 600 MHz, CD_3OD).

Figure S6. NOESY spectrum of compound **1** (TFA salt, 600 MHz, CD_3OD).

Figure S7. HR-ESIMS spectrum of compound **1**.

Figure S8. Experimental ECD spectra (raw) of compounds **1, 4**, **5**, and **6** (TFA salt) in CH_3OH.

Figure S9. ^{1}H NMR spectrum of compound **2** (TFA salt, 600 MHz, DMSO-*d*6).

Figure S10. HSQC spectrum of compound **2** (TFA salt, 600 MHz, DMSO-*d*6).

Figure S11. HMBC spectrum of compound **2** (TFA salt, 600 MHz, DMSO-*d*6).

Figure S12. COSY spectrum of compound **2** (TFA salt, 600 MHz, DMSO-*d*6).

Figure S13. NOESY spectrum of compound **2** (TFA salt, 600 MHz, DMSO-*d*6).

Figure S14. HR-ESIMS spectrum of compound **2**.

Figure S15. ^{1}H NMR spectrum of compound **3** (TFA salt, 600 MHz, CD_3OD).

Figure S16. HSQC spectrum of compound **3** (TFA salt, 600 MHz, CD_3OD).

Figure S17. HMBC spectrum of compound **3** (TFA salt, 600 MHz, CD_3OD).

Figure S18. COSY spectrum of compound **3** (TFA salt, 600 MHz, CD_3OD).

Figure S19. NOESY spectrum of compound **3** (TFA salt, 600 MHz, CD_3OD).

Figure S20. ^{1}H NMR spectrum of compound **3** (TFA salt, 600 MHz, Acetone-d_6).

Figure S21. HR-ESIMS spectrum of compound **3**.

Figure S22. ^{1}H NMR spectrum of compound **4** (TFA salt, 600 MHz, CD_3OD).

Figure S23. HSQC spectrum of compound **4** (TFA salt, 600 MHz, CD_3OD).

Figure S24. HMBC spectrum of compound **4** (TFA salt, 600 MHz, CD_3OD).

Figure S25. COSY spectrum of compound **4** (TFA salt, 600 MHz, CD_3OD).

Figure S26. NOESY spectrum of compound **4** (TFA salt, 600 MHz, CD_3OD).

Figure S27. HR-ESIMS spectrum of compound **4**.

Figure S28. ^1H NMR spectrum of compound **5** (TFA salt, 600 MHz, CD_3OD).

Figure S29. ^{13}C NMR spectrum of compound **5** (TFA salt, 150 MHz, CD_3OD).

Figure S30. HSQC spectrum of compound **5** (TFA salt, 600 MHz, CD_3OD).

Figure S31. HMBC spectrum of compound **5** (TFA salt, 600 MHz, CD_3OD).

Figure S32. COSY spectrum of compound **5** (TFA salt, 600 MHz, CD_3OD).

Figure S33. NOESY spectrum of compound **5** (TFA salt, 600 MHz, CD_3OD).

Figure S34. HR-ESIMS spectrum of compound **5**.

Figure S35. ^1H NMR spectrum of compound **6** (TFA salt, 600 MHz, CD_3OD).

Figure S36. ^{13}C NMR spectrum of compound **6** (TFA salt, 150 MHz, CD_3OD).

Figure S37. HSQC spectrum of compound **6** (TFA salt, 600 MHz, CD_3OD).

Figure S38. HMBC spectrum of compound **6** (TFA salt, 600 MHz, CD_3OD).

Figure S39. COSY spectrum of compound **6** (TFA salt, 600 MHz, CD_3OD).

Figure S40. NOESY spectrum of compound **6** (TFA salt, 600 MHz, CD_3OD).

Figure S41. HR-ESIMS spectrum of compound **6**.

Figure S1. ^{1}H NMR spectrum of compound **1** (TFA salt, 600 MHz, CD_3OD).

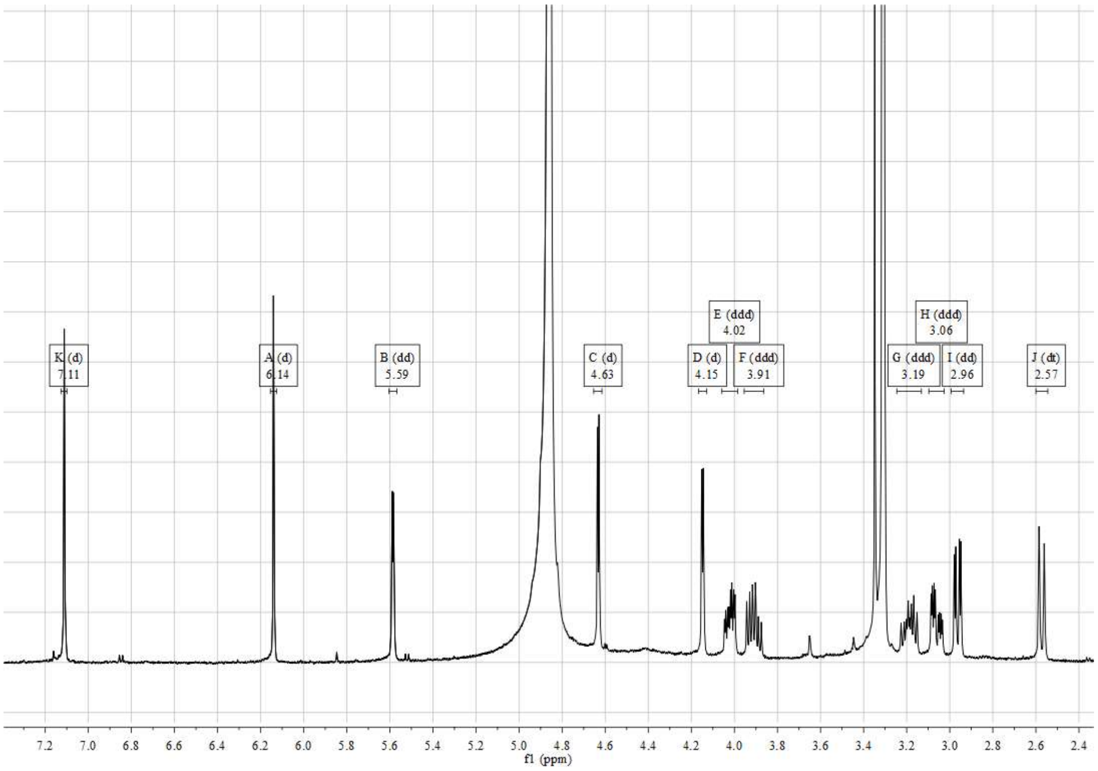

Figure S2. ^{13}C NMR spectrum of compound **1** (TFA salt, 150 MHz, CD_3OD).

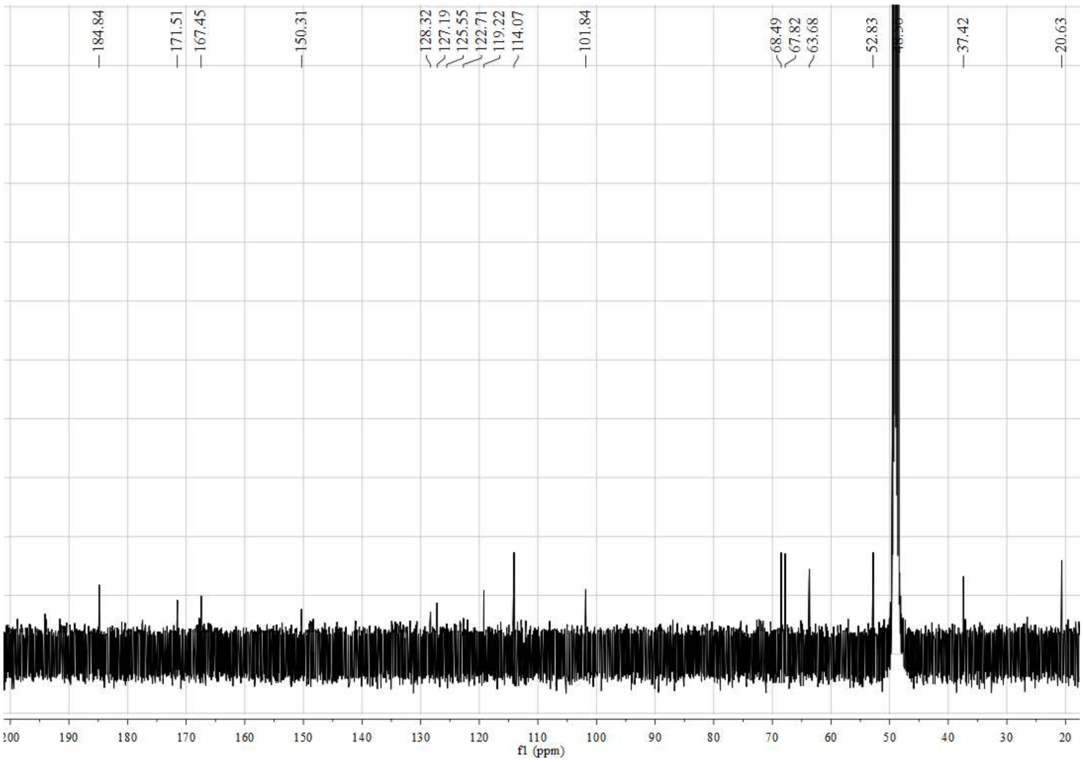

Figure S3. HSQC spectrum of compound **1** (TFA salt, 600 MHz, CD3OD).

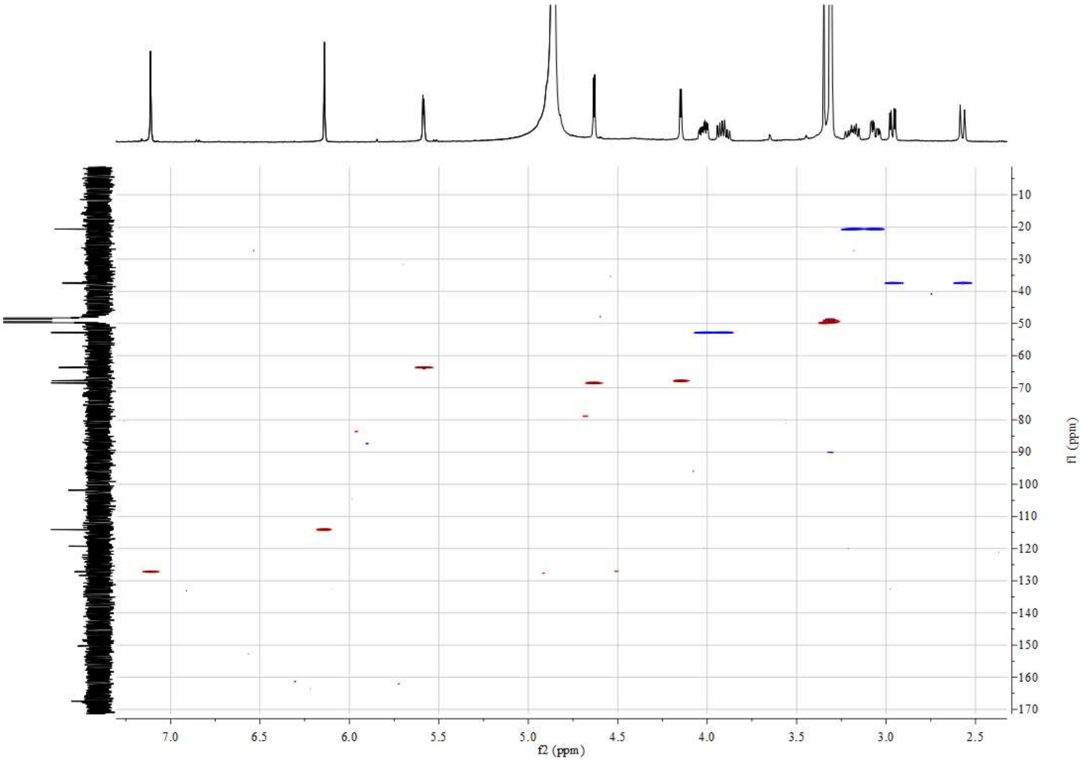

Figure S4. HMBC spectrum of compound **1** (TFA salt, 600 MHz, CD3OD).

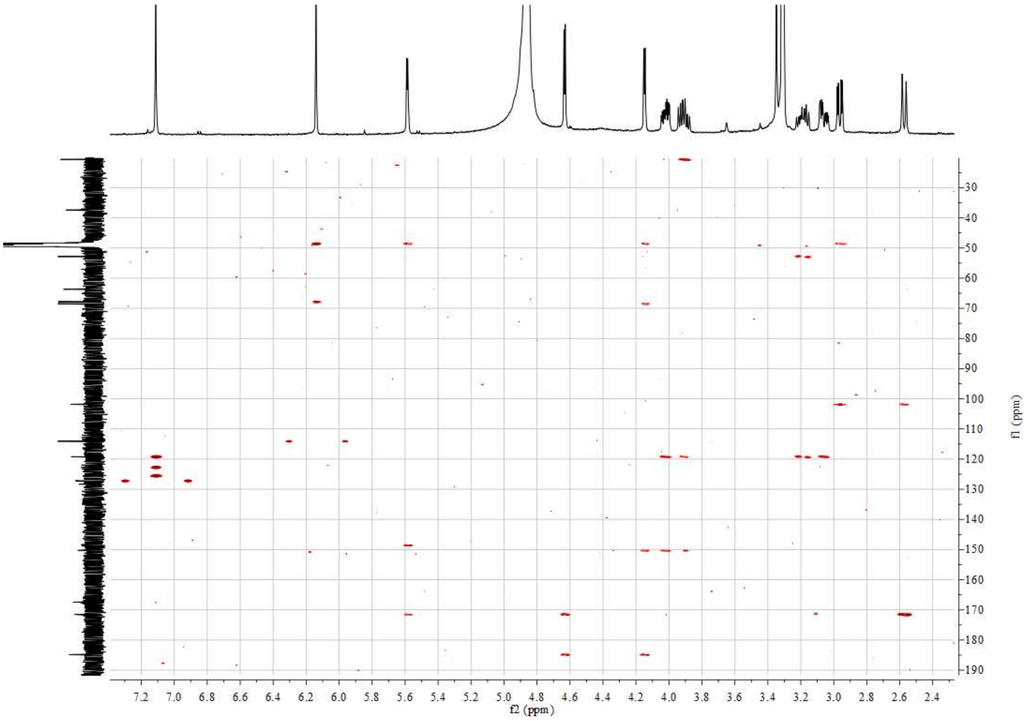

Figure S5. COSY spectrum of compound **1** (TFA salt, 600 MHz, CD_3OD).

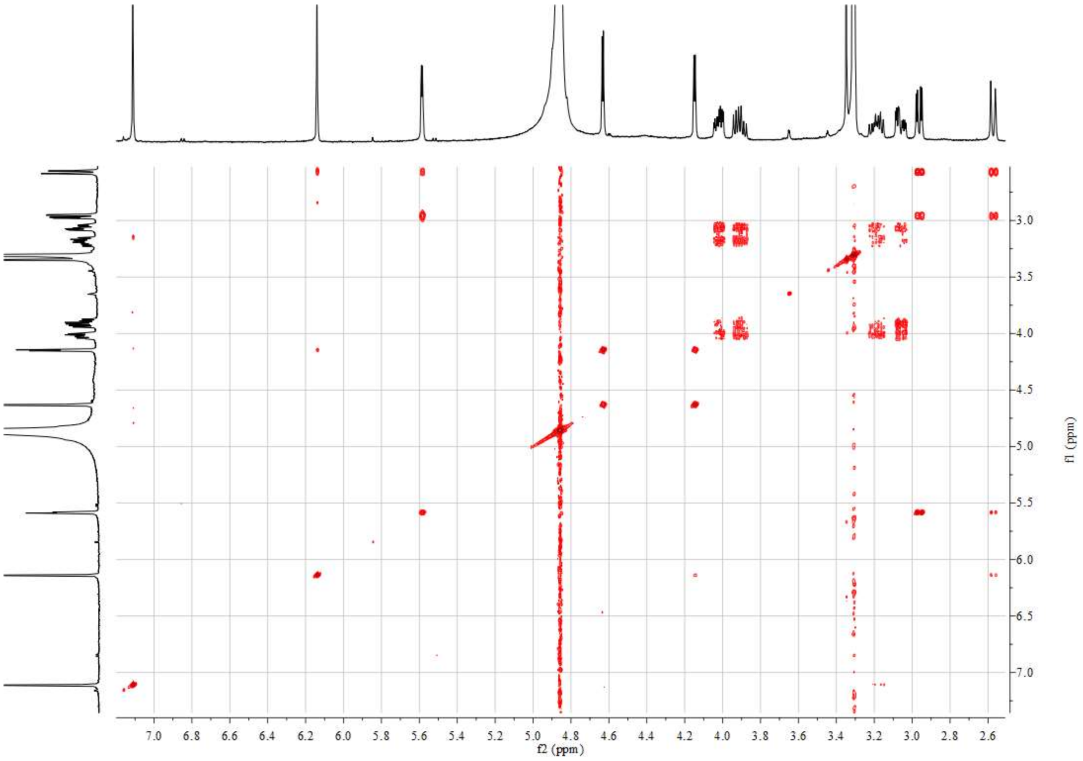

Figure S6. NOESY spectrum of compound **1** (TFA salt, 600 MHz, CD_3OD).

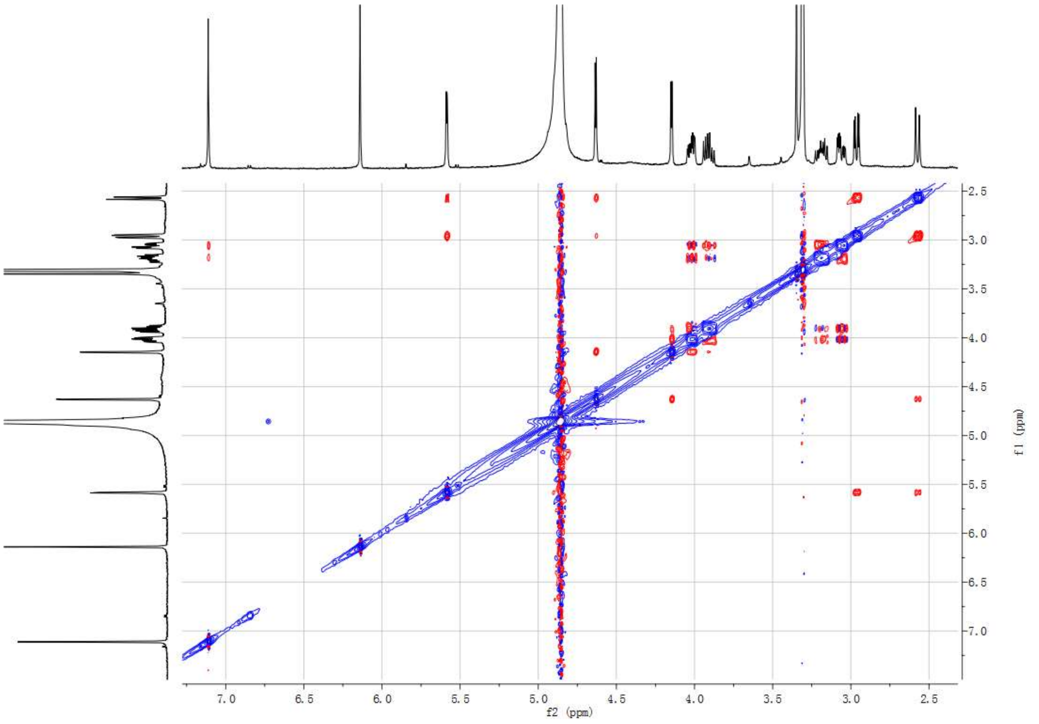

Figure S7. HR-ESIMS spectrum of compound **1**.

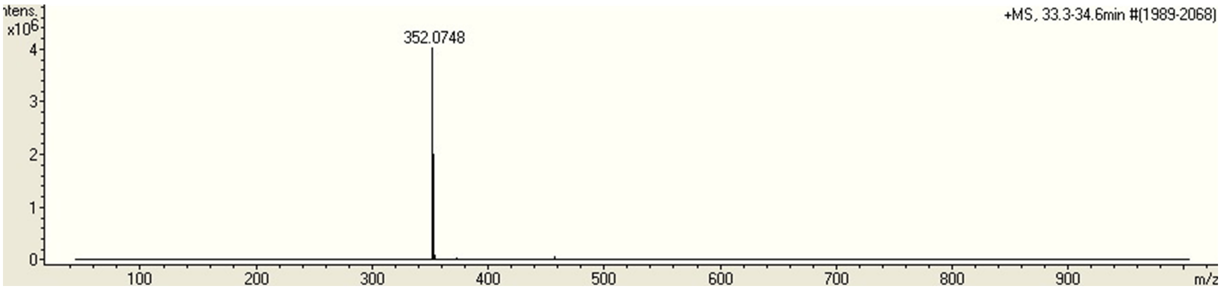

Figure S8. Experimental ECD spectra (raw) of compounds **1, 4**, **5**, and **6** (TFA salt) in CH_3OH.

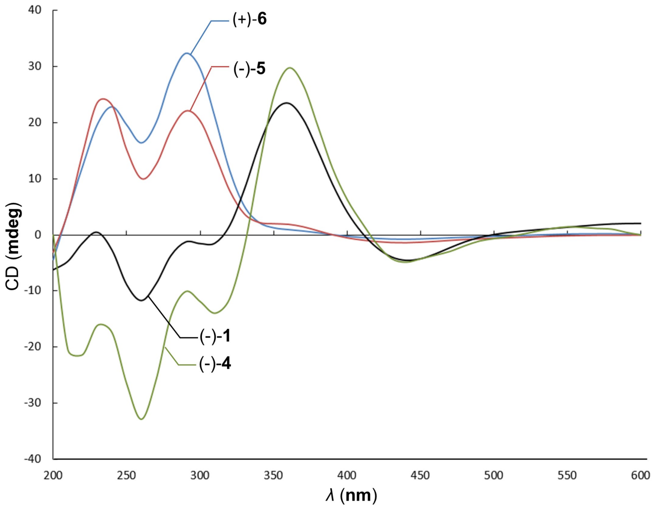

Figure S9. ^{1}H NMR spectrum of compound **2** (TFA salt, 600 MHz, DMSO-*d*6).

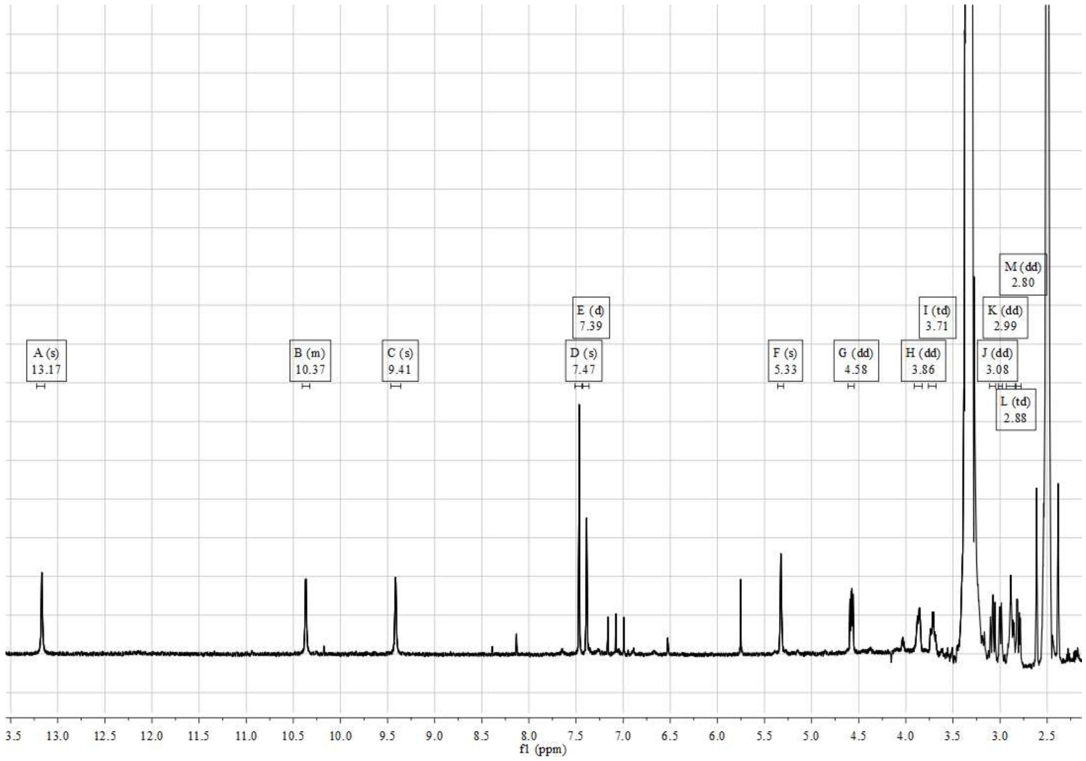

Figure S10. HSQC spectrum of compound **2** (TFA salt, 600 MHz, DMSO-*d*6).

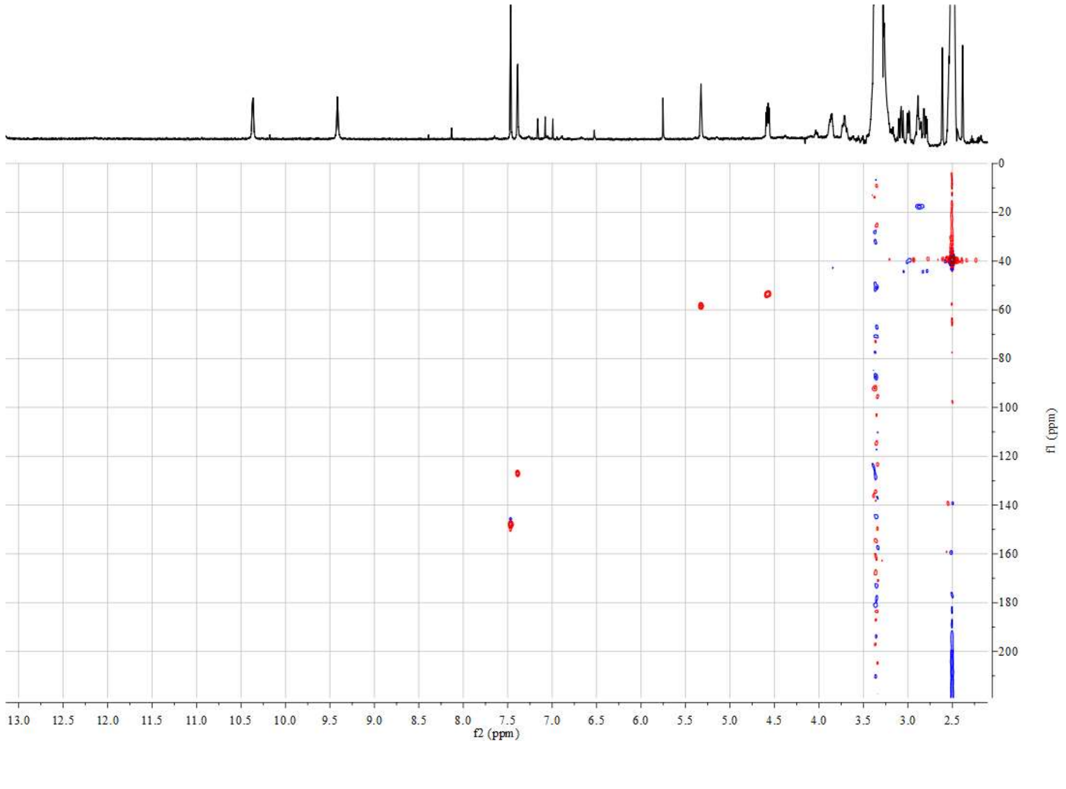

Figure S11. HMBC spectrum of compound **2** (TFA salt, 600 MHz, DMSO-*d*6).

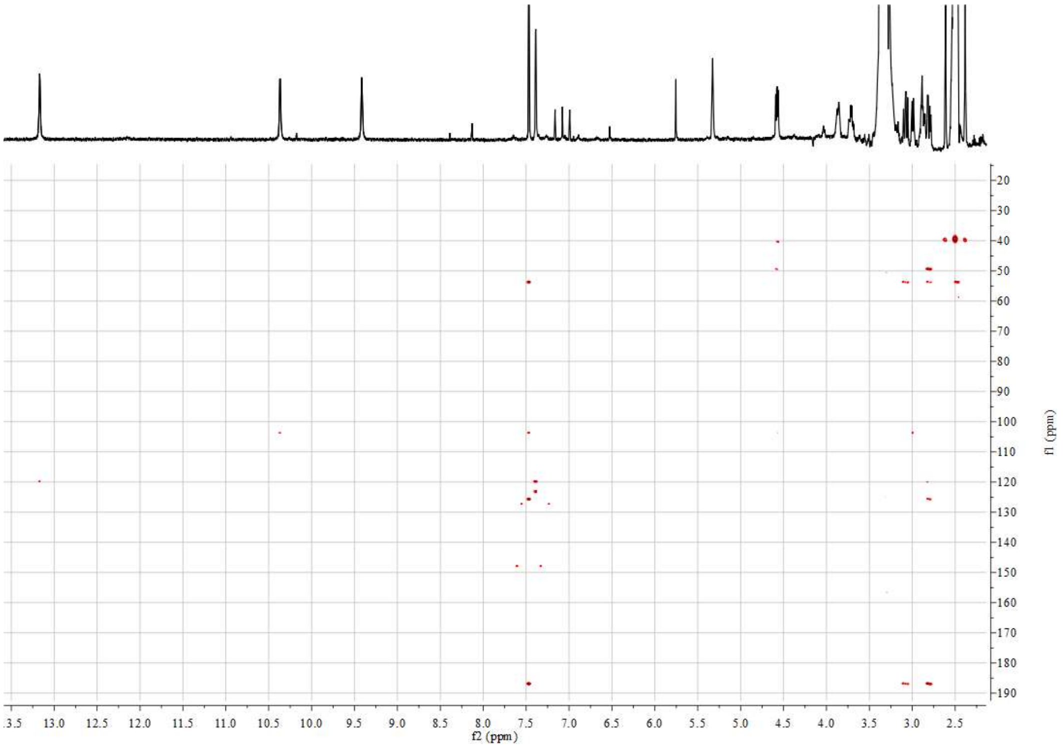

Figure S12. COSY spectrum of compound **2** (TFA salt, 600 MHz, DMSO-*d*6).

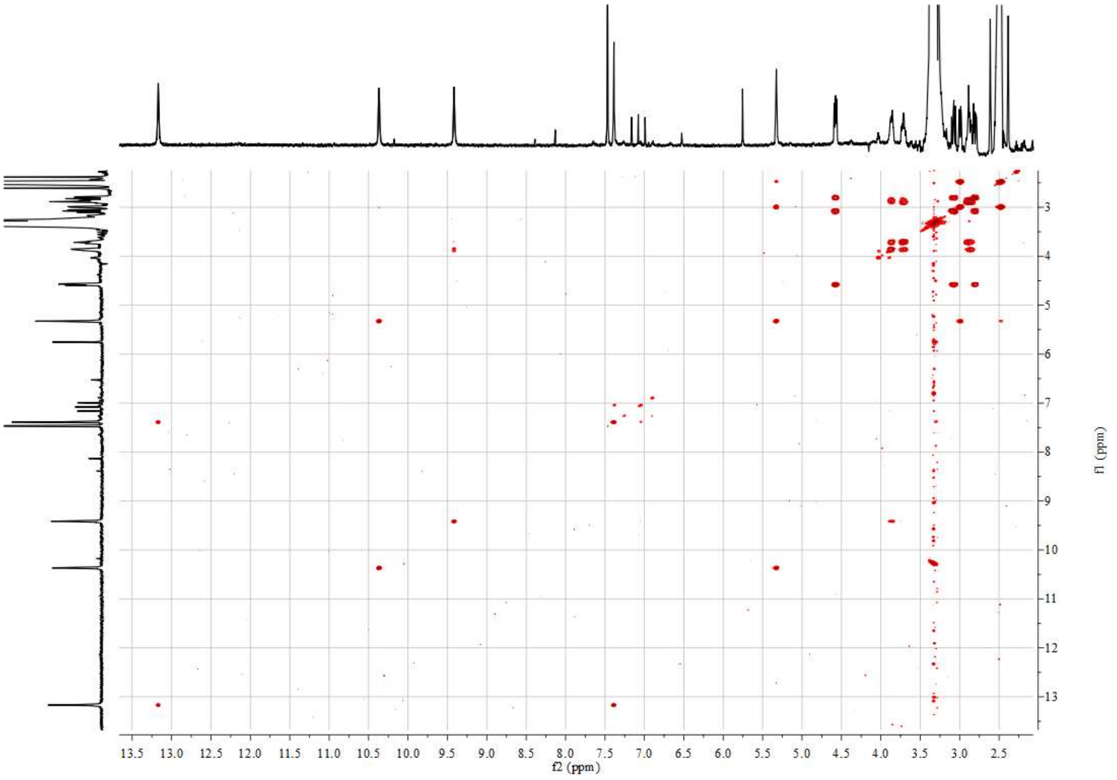

Figure S13. NOESY spectrum of compound **2** (TFA salt, 600 MHz, DMSO-*d6*).

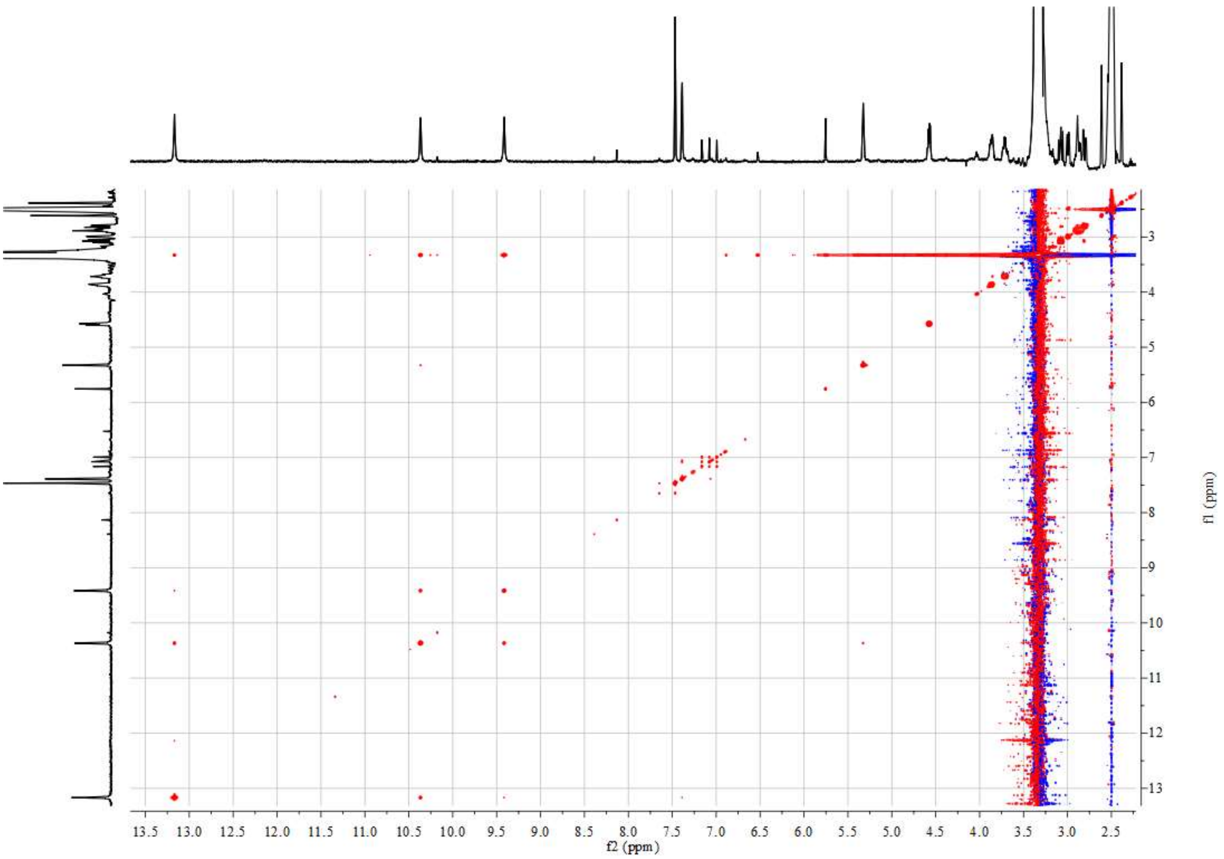

Figure S14. HR-ESIMS spectrum of compound **2**.

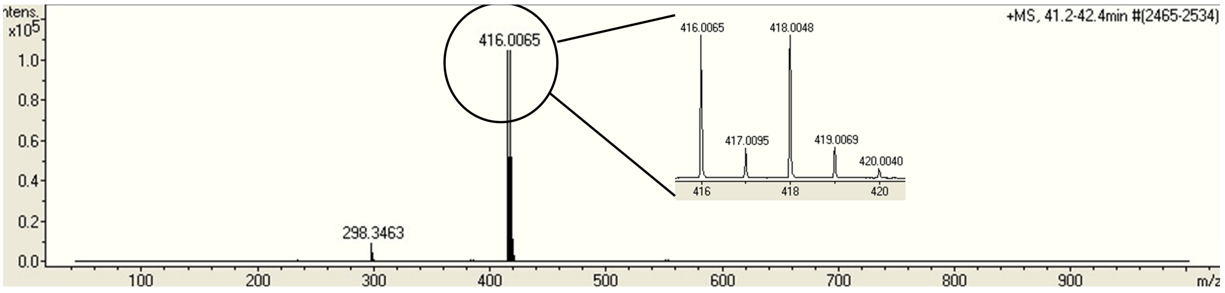

Figure S15. ^{1}H NMR spectrum of compound **3** (TFA salt, 600 MHz, CD_3OD).

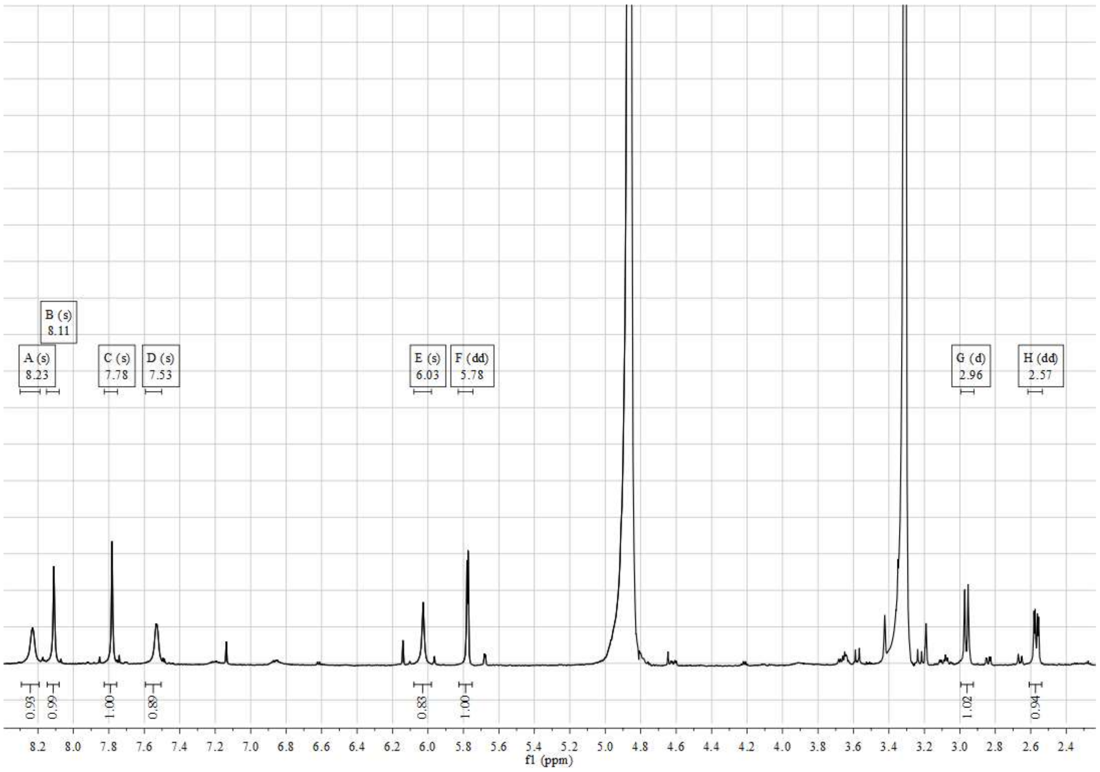

Figure S16. HSQC spectrum of compound **3** (TFA salt, 600 MHz, CD_3OD).

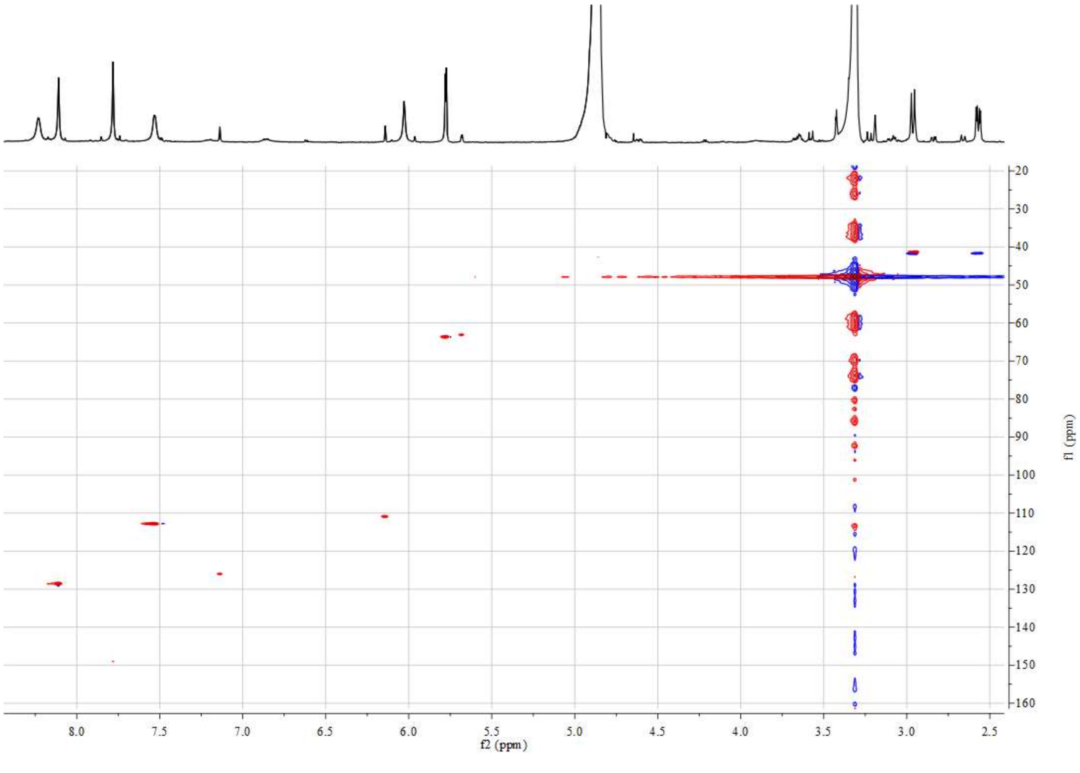

Figure S17. HMBC spectrum of compound **3** (TFA salt, 600 MHz, CD_3OD).

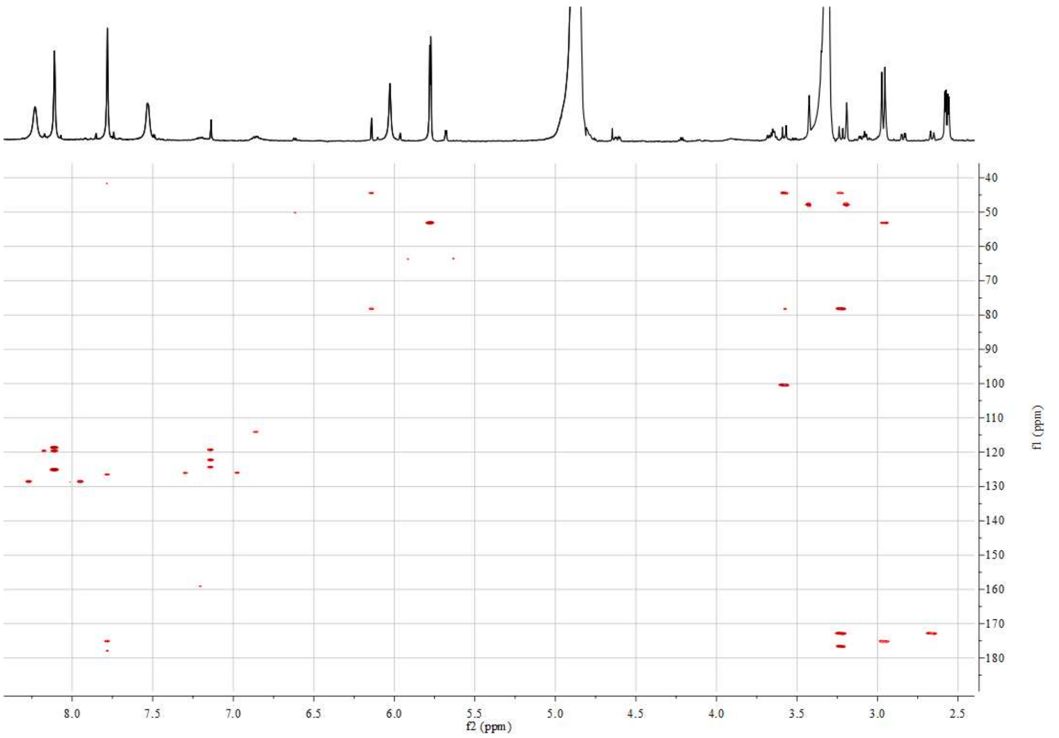

Figure S18. COSY spectrum of compound **3** (TFA salt, 600 MHz, CD_3OD).

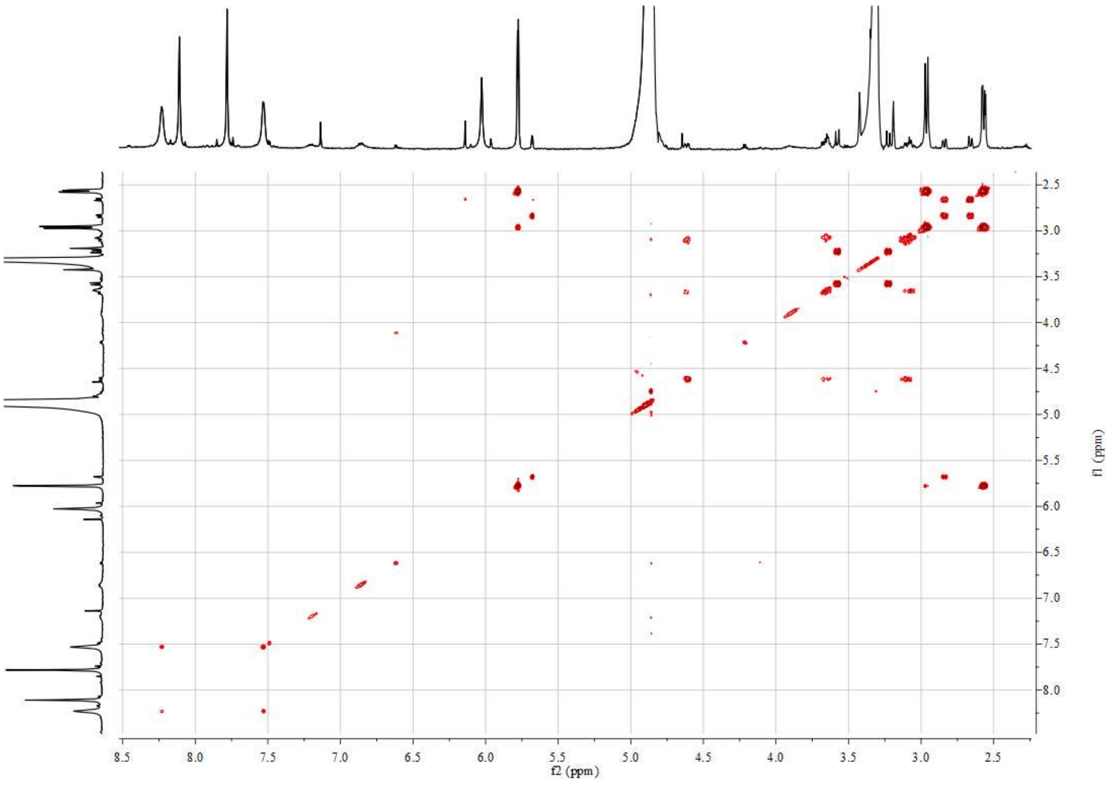

Figure S19. NOESY spectrum of compound **3** (TFA salt, 600 MHz, CD_3OD).

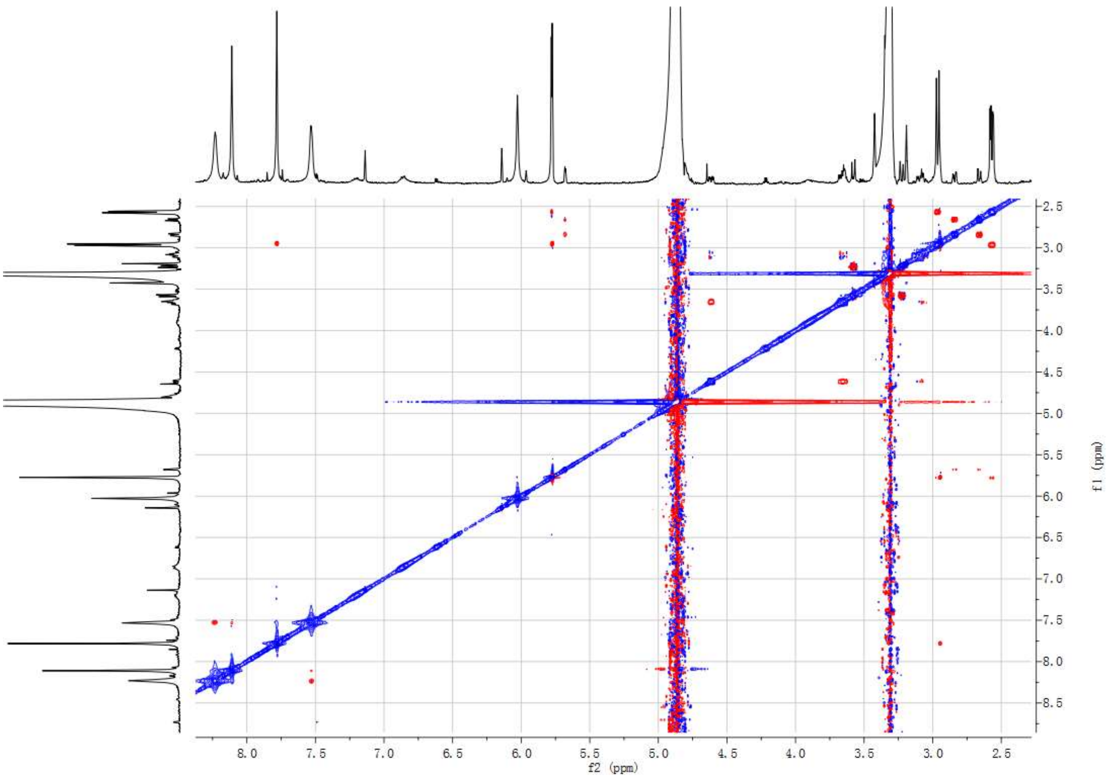

Figure S20. ^1H NMR spectrum of compound **3** (TFA salt, 600 MHz, Acetone- d_6).

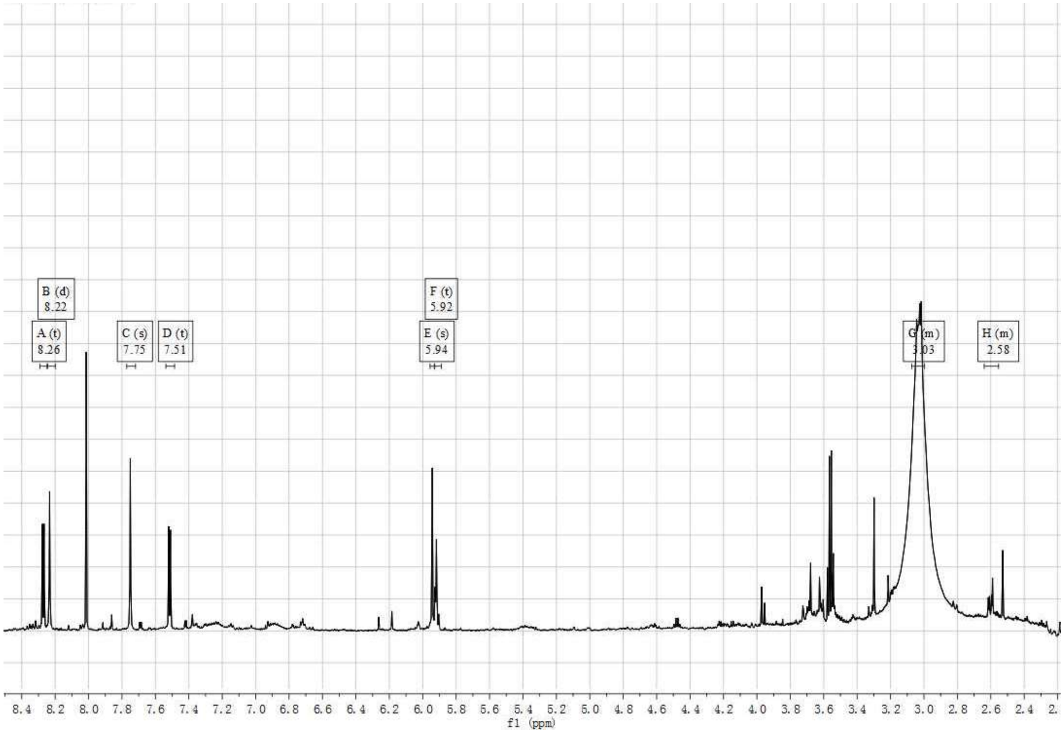

Figure S21. HR-ESIMS spectrum of compound **3**.

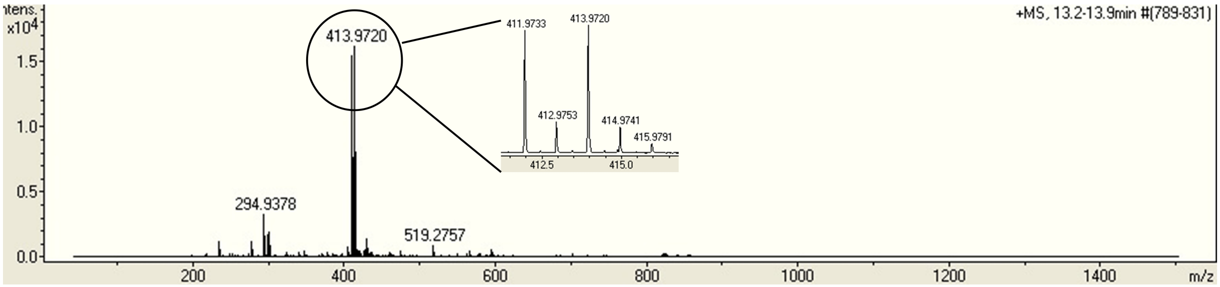

Figure S22. ^{1}H NMR spectrum of compound **4** (TFA salt, 600 MHz, CD_3OD).

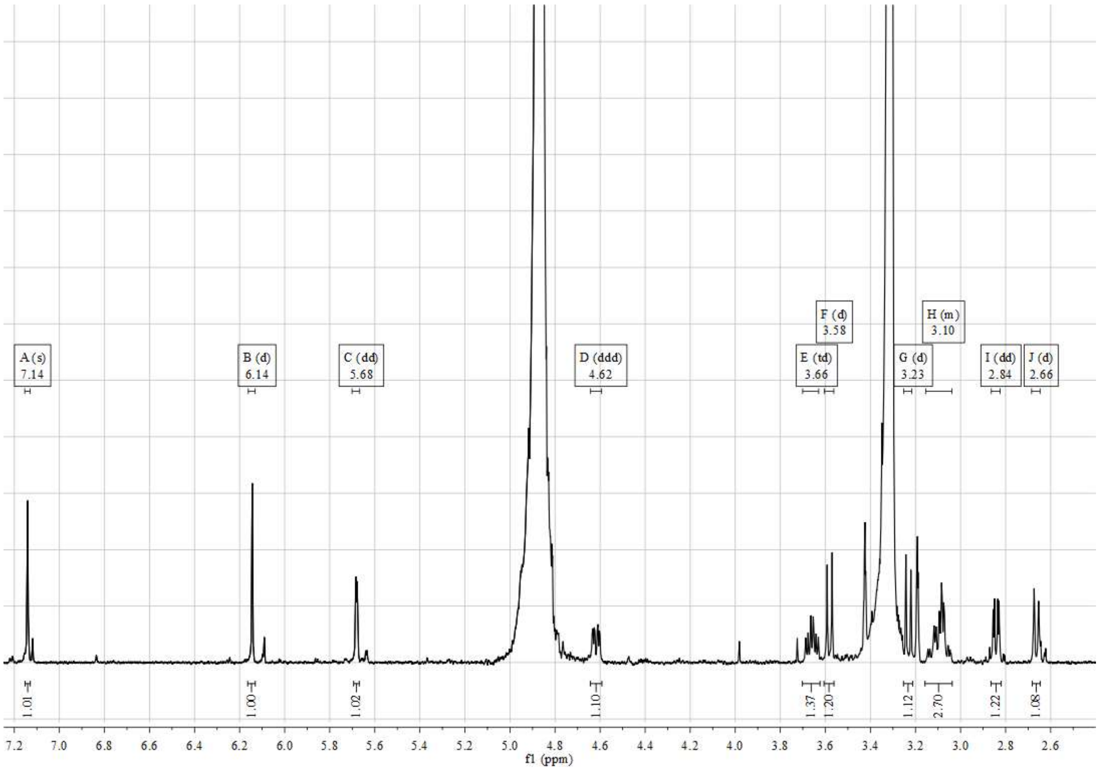

Figure S23. HSQC spectrum of compound **4** (TFA salt, 600 MHz, CD_3OD).

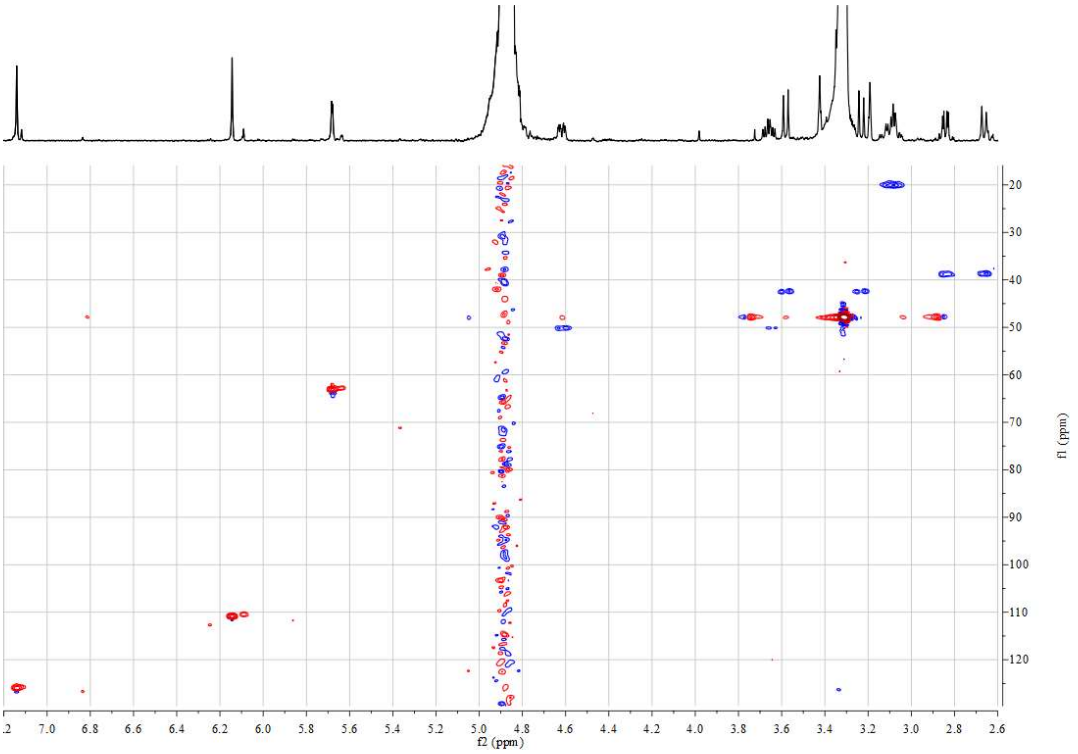

Figure S24. HMBC spectrum of compound **4** (TFA salt, 600 MHz, CD_3OD).

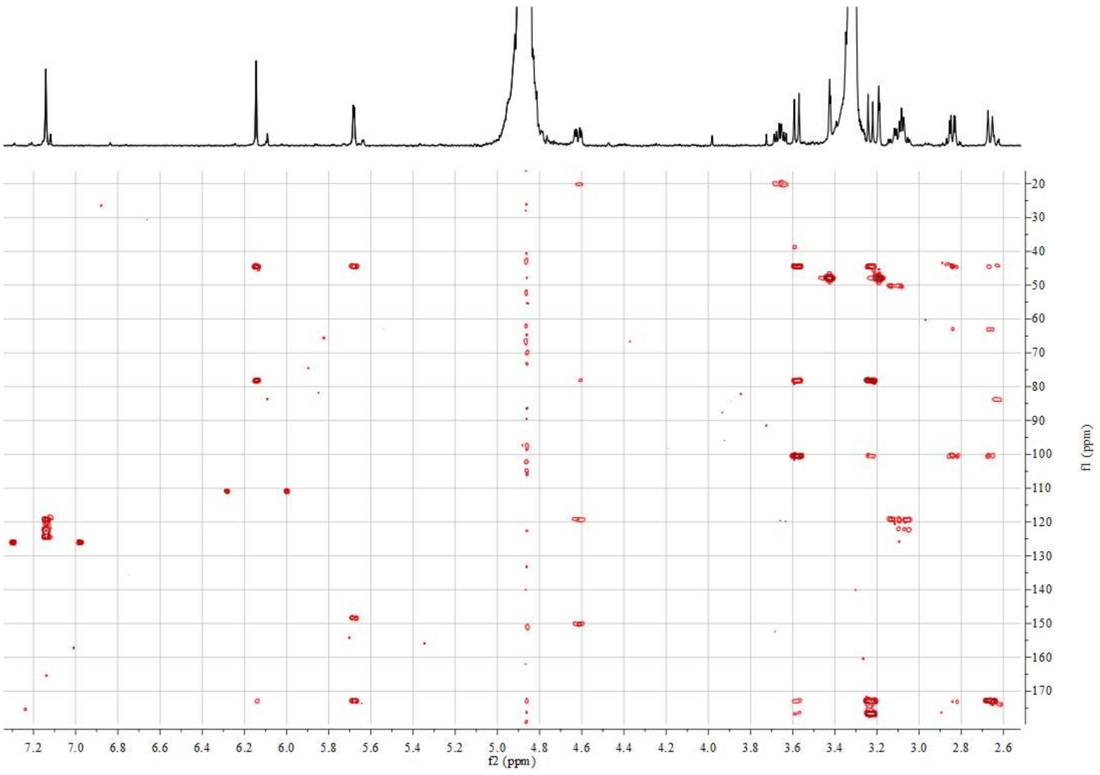

Figure S25. COSY spectrum of compound **4** (TFA salt, 600 MHz, CD_3OD).

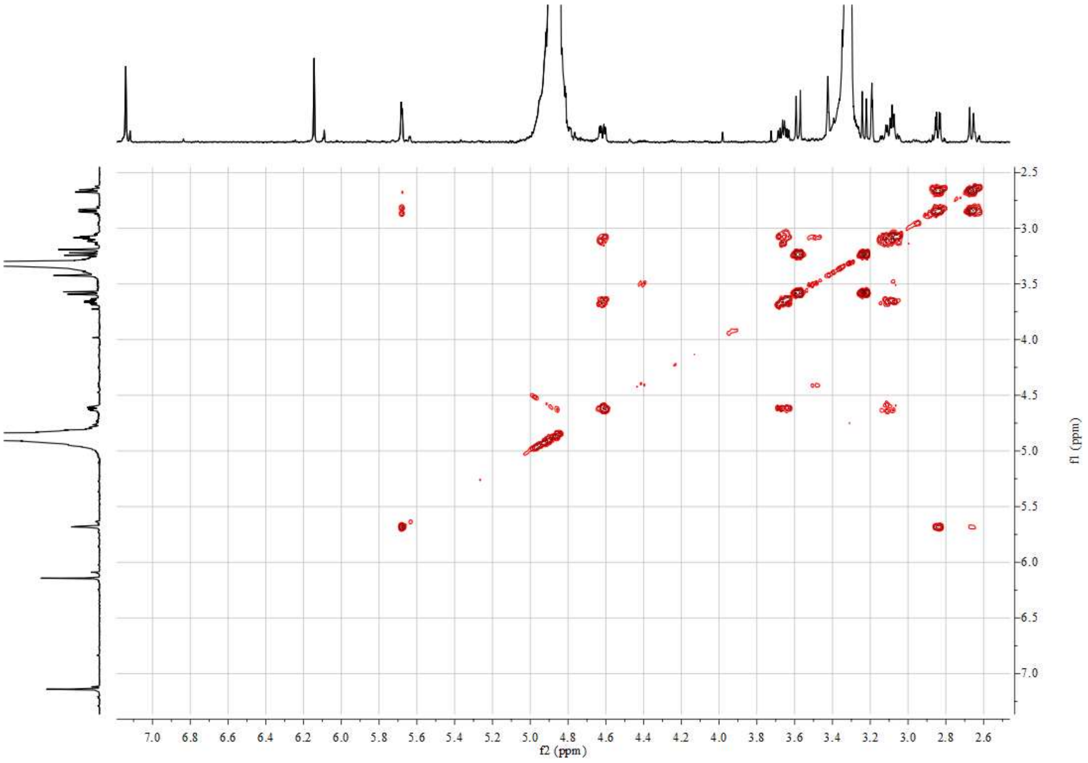

Figure S26. NOESY spectrum of compound **4** (TFA salt, 600 MHz, CD_3OD).

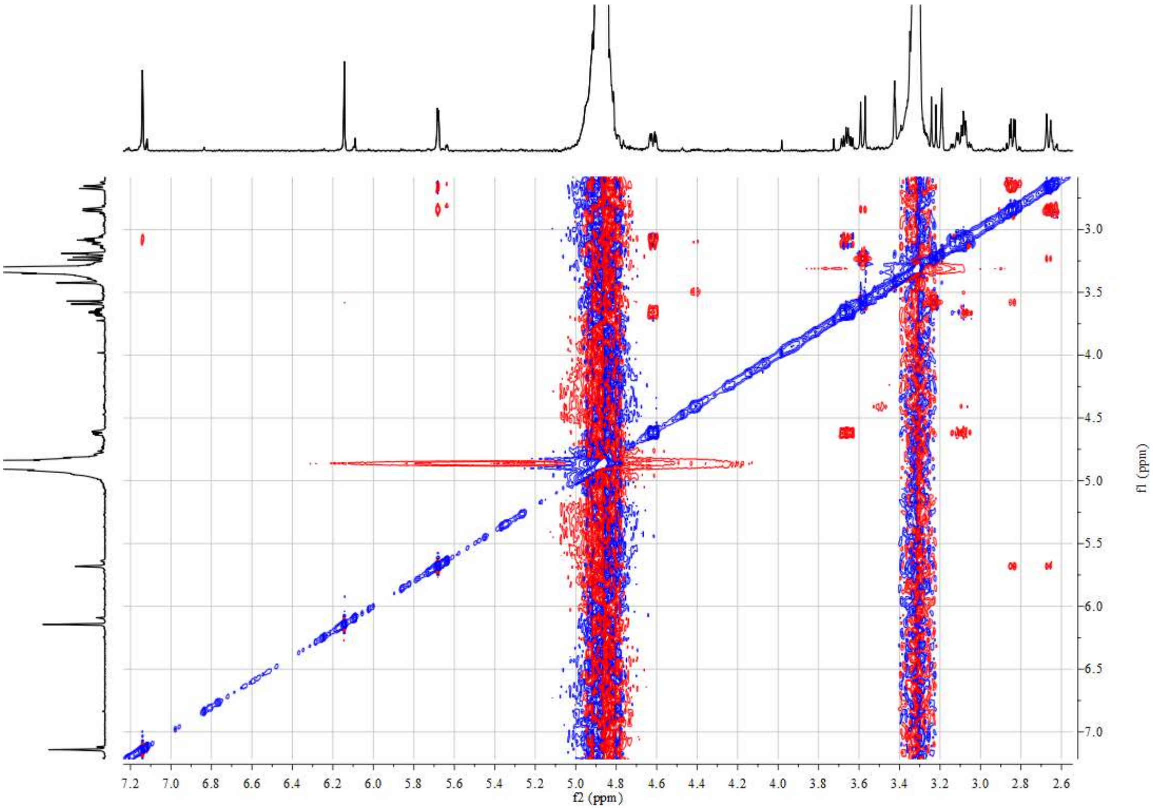

Figure S27. HR-ESIMS spectrum of compound **4**.

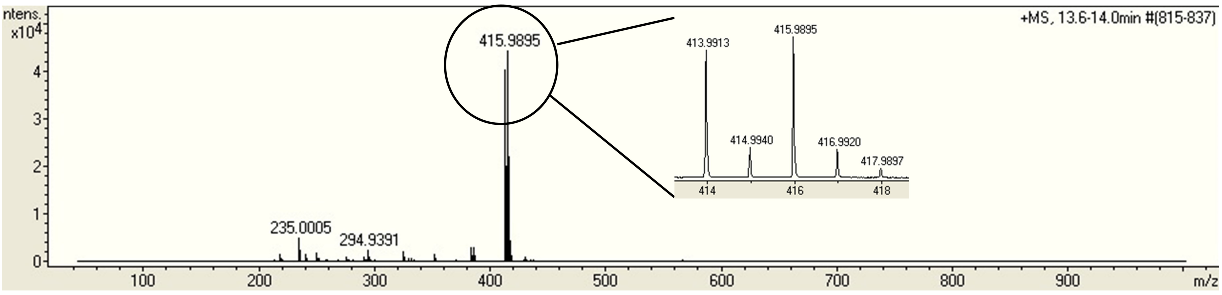

Figure S28. ^{1}H NMR spectrum of compound **5** (TFA salt, 600 MHz, CD_3OD).

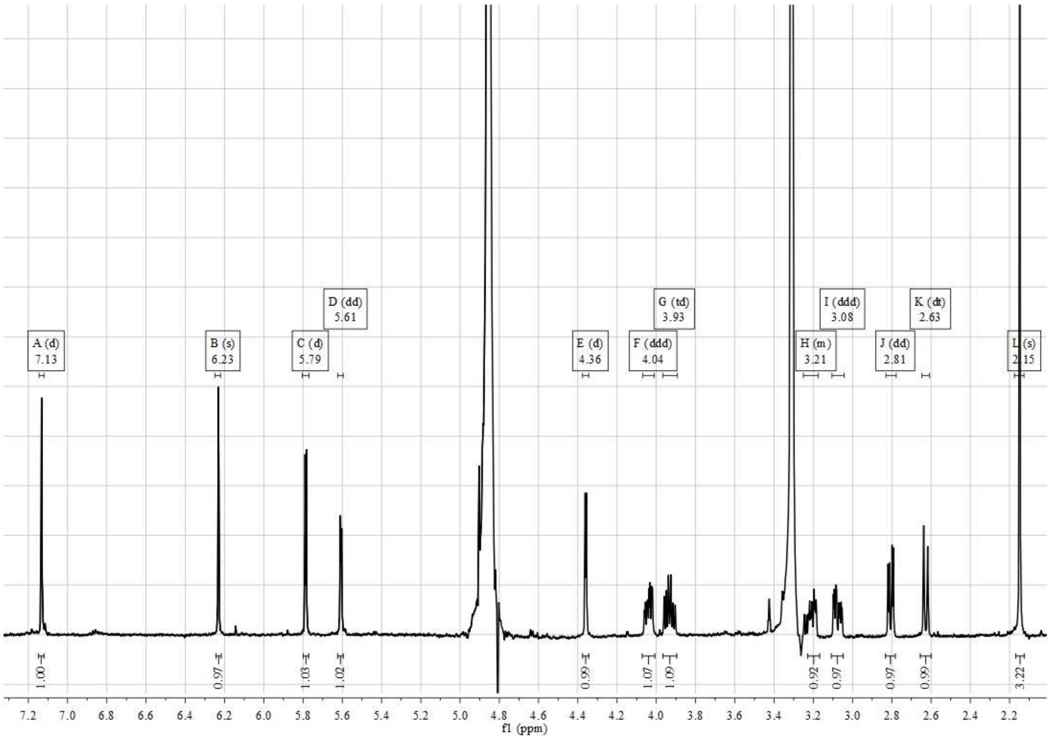

Figure S29. ^{13}C NMR spectrum of compound **5** (TFA salt, 150 MHz, CD_3OD).

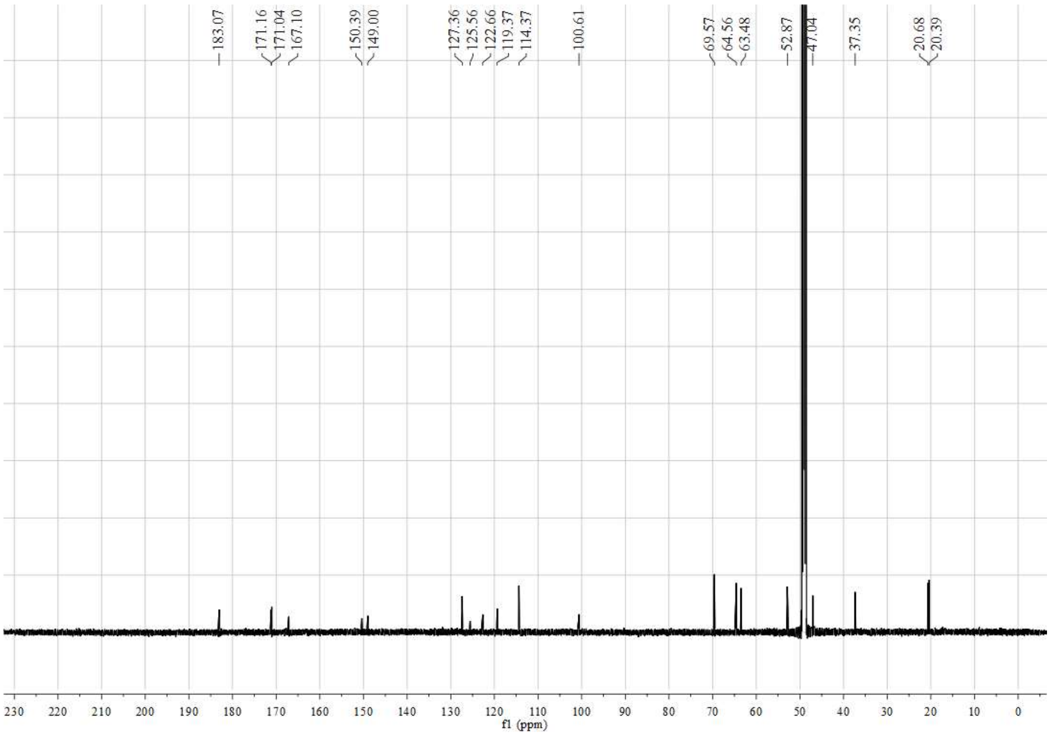

Figure S30. HSQC spectrum of compound **5** (TFA salt, 600 MHz, CD_3OD).

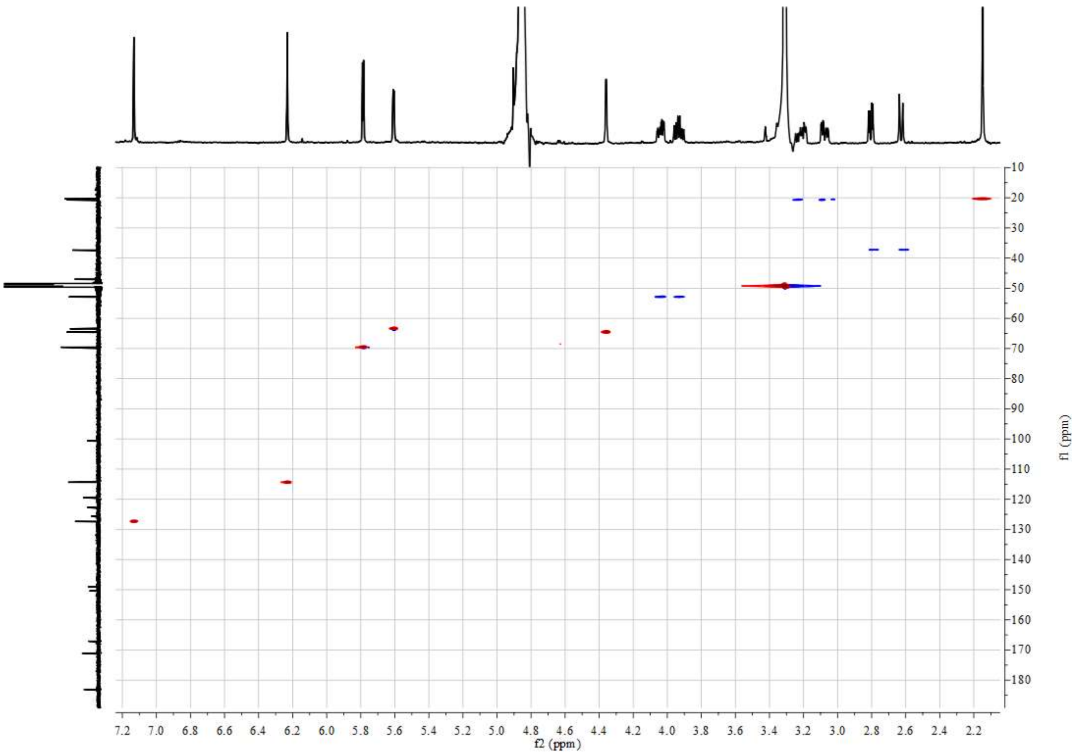

Figure S31. HMBC spectrum of compound **5** (TFA salt, 600 MHz, CD_3OD).

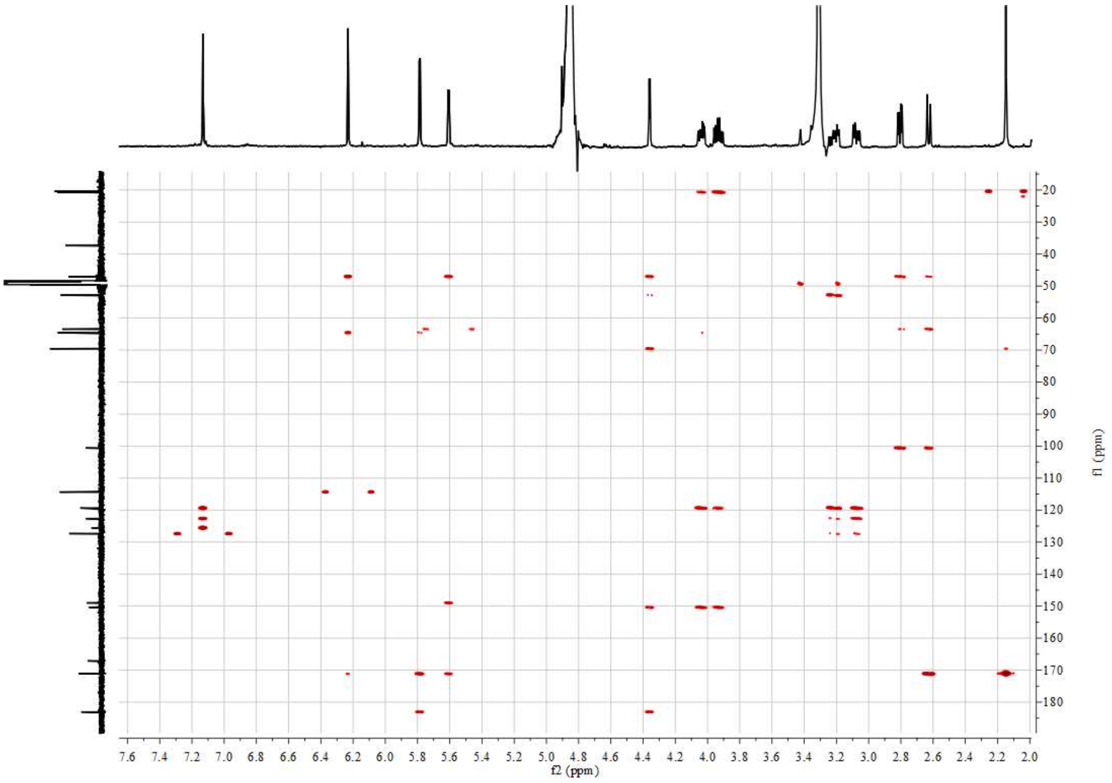

Figure S32. COSY spectrum of compound **5** (TFA salt, 600 MHz, CD_3OD).

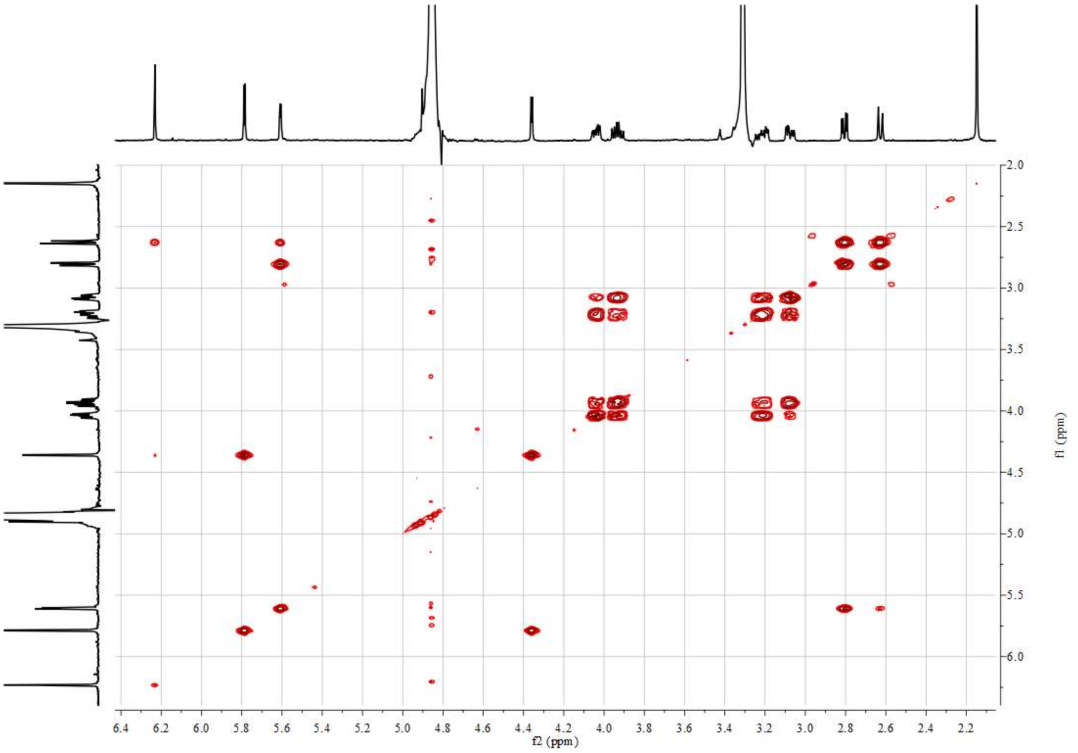

Figure S33. NOESY spectrum of compound **5** (TFA salt, 600 MHz, CD_3OD).

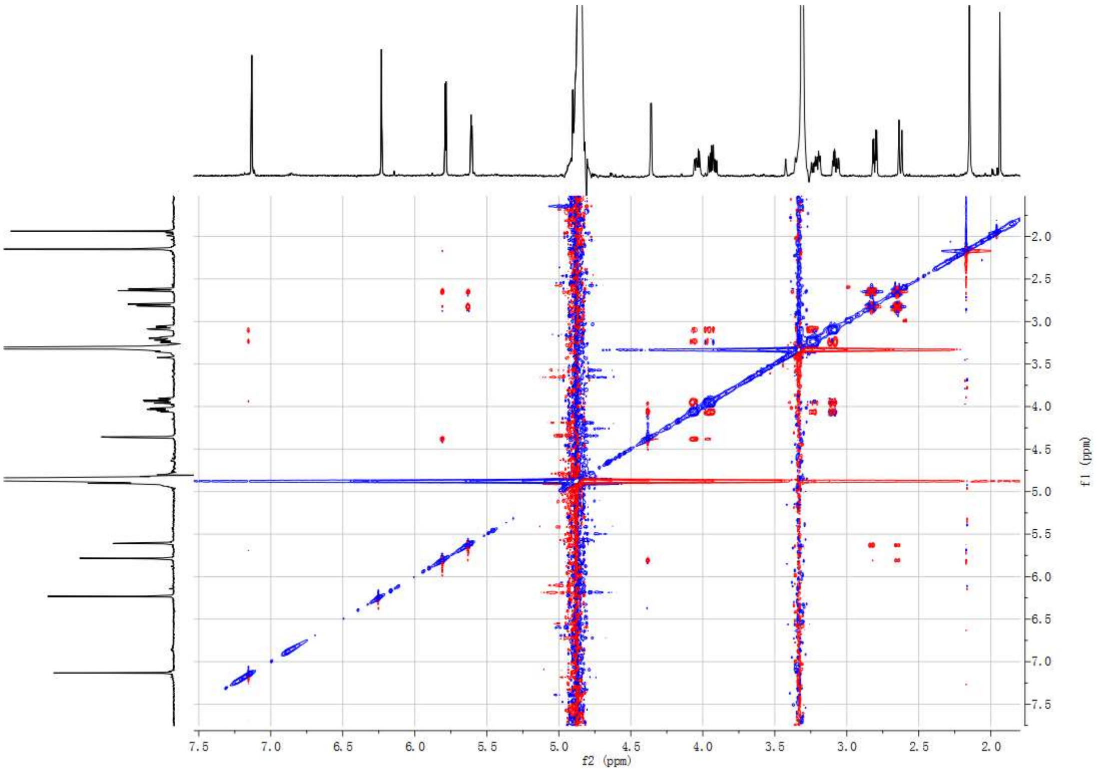

Figure S34. HR-ESIMS spectrum of compound **5**.

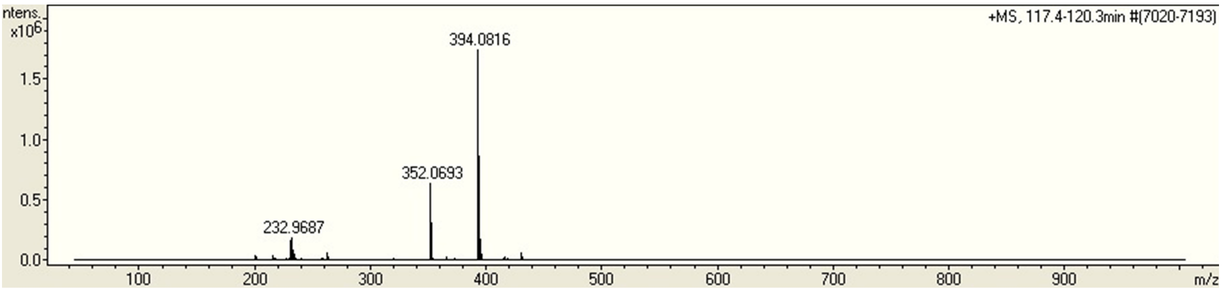

Figure S35. ^{1}H NMR spectrum of compound **6** (TFA salt, 600 MHz, CD_3OD).

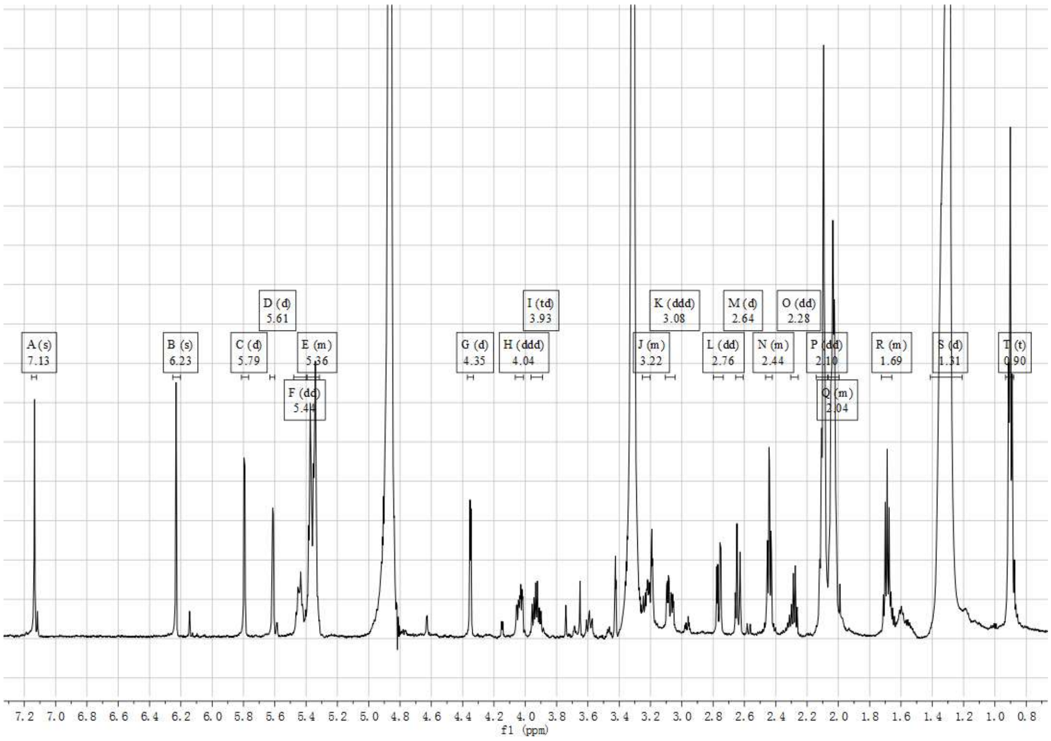

Figure S36. ^{13}C NMR spectrum of compound **6** (TFA salt, 150 MHz, CD_3OD).

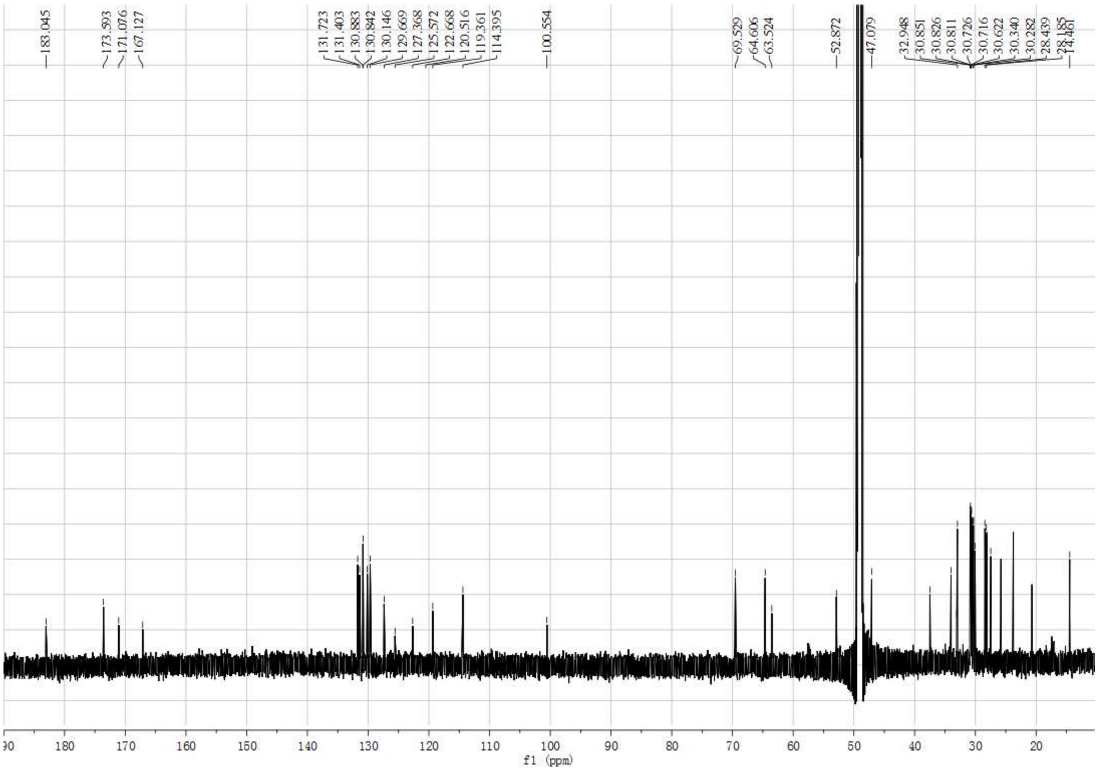

Figure S37. HSQC spectrum of compound **6** (TFA salt, 600 MHz, CD3OD).

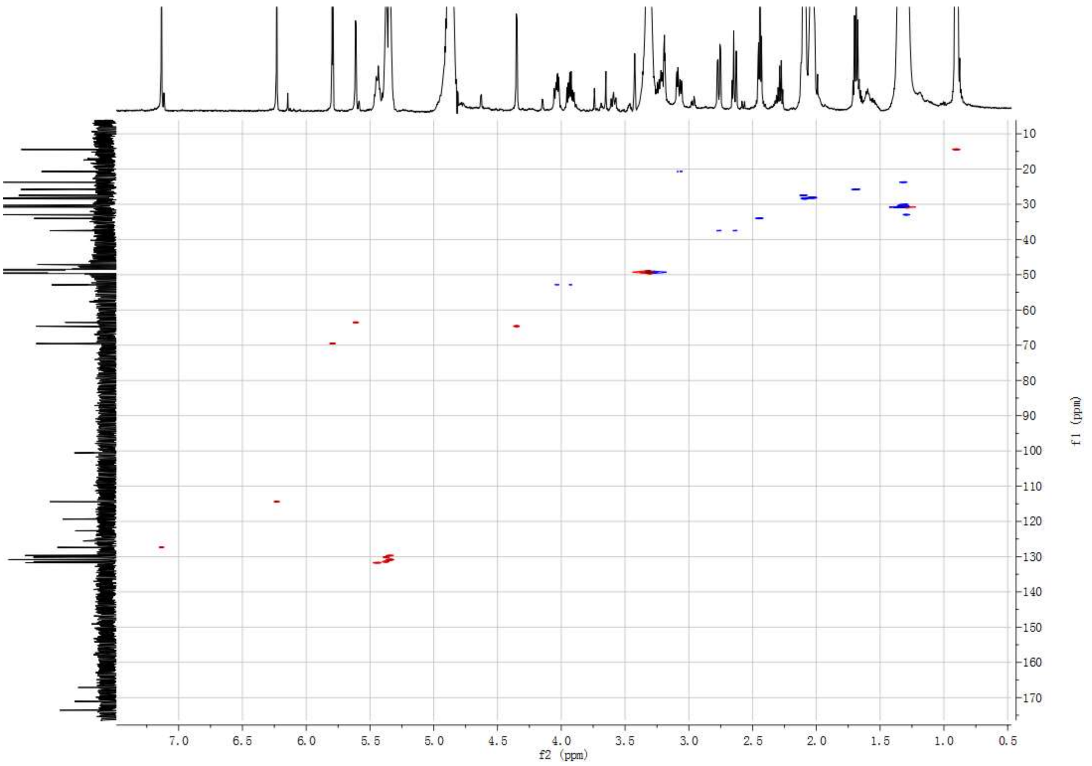

Figure S38. HMBC spectrum of compound **6** (TFA salt, 600 MHz, CD3OD).

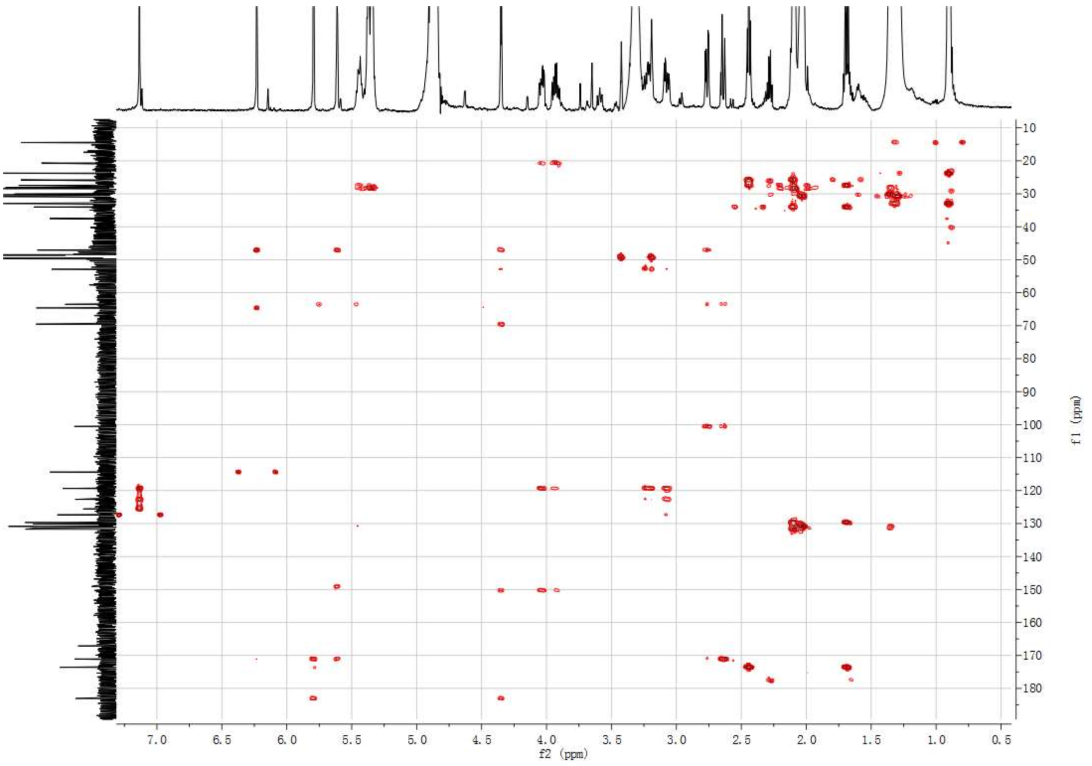

Figure S39. COSY spectrum of compound **6** (TFA salt, 600 MHz, CD_3OD).

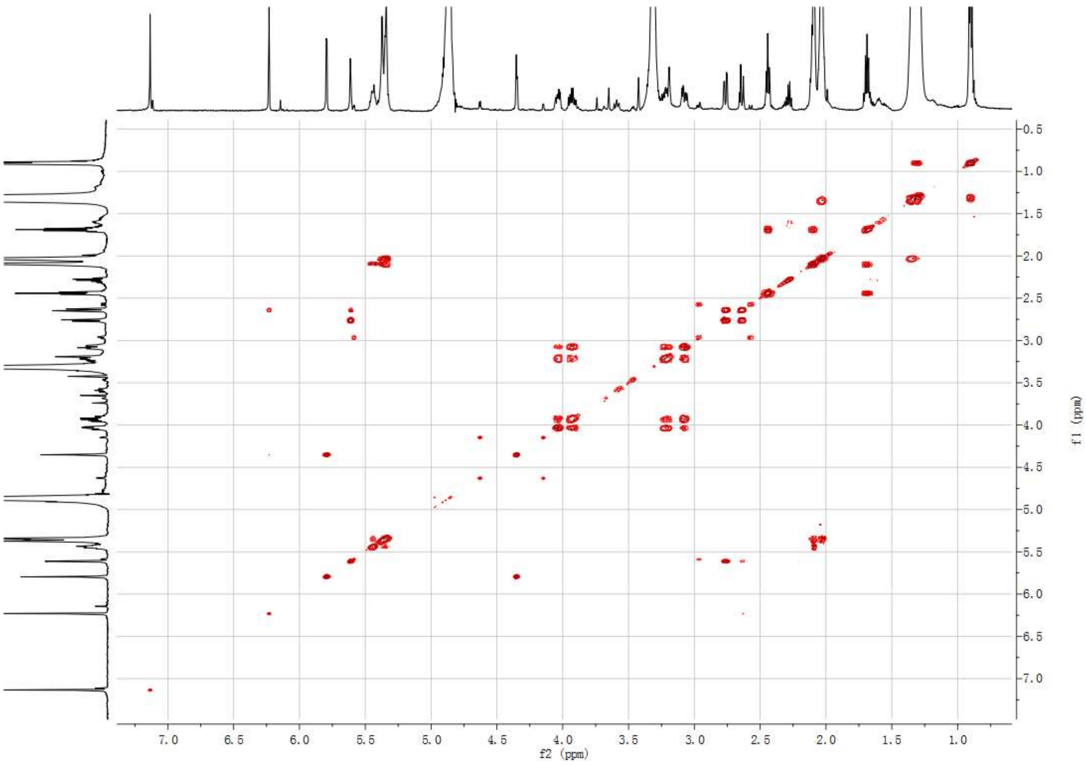

Figure S40. NOESY spectrum of compound **6** (TFA salt, 600 MHz, CD_3OD).

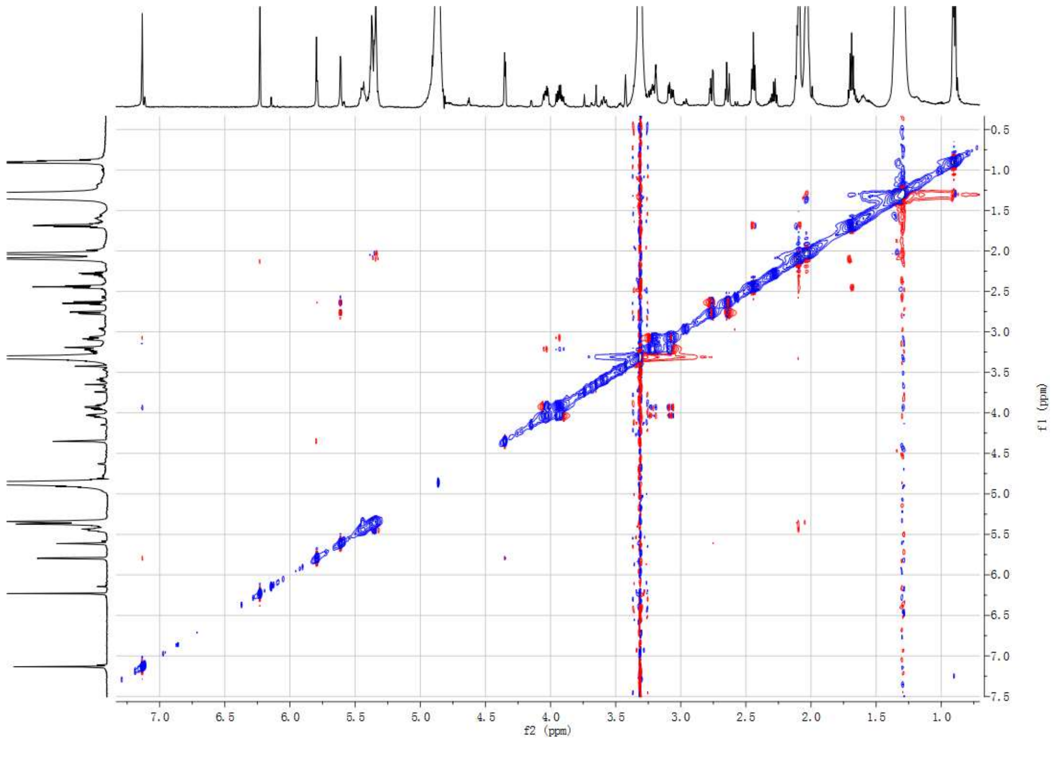

Figure S41. HR-ESIMS spectrum of compound **6**.

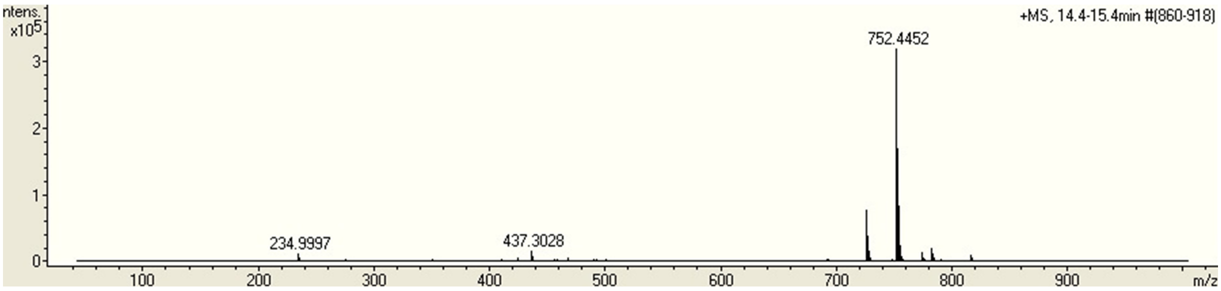

Supplement: Supplementary file 1 [file marinedrugs-17-00439-s001.pdf]
